# Atmospheric Correction for High-Resolution Shape from Shading on Mars

**DOI:** 10.3390/jimaging8060158

**Published:** 2022-06-01

**Authors:** Marcel Hess, Moritz Tenthoff, Kay Wohlfarth, Christian Wöhler

**Affiliations:** Image Analysis Group, TU Dortmund University, Otto-Hahn-Str. 4, 44227 Dortmund, Germany; moritz.tenthoff@tu-dortmund.de (M.T.); kay.wohlfarth@tu-dortmund.de (K.W.); christian.woehler@tu-dortmund.de (C.W.)

**Keywords:** Mars, shape from shading, atmosphere, digital elevation model, Hapke model

## Abstract

Digital Elevation Models (DEMs) of planet Mars are crucial for many remote sensing applications and for landing site characterization of rover missions. Shape from Shading (SfS) is known to work well as a complementary method to greatly enhance the quality of photogrammetrically obtained DEMs of planetary surfaces with respect to the effective resolution and the overall accuracy. In this work, we extend our previous lunar shape and albedo from shading framework by embedding the Hapke photometric reflectance model in an atmospheric model such that it is applicable to Mars. Compared to previous approaches, the proposed method is capable of directly estimating the atmospheric parameters from a given scene without the need for external data, and assumes a spatially varying albedo. The DEMs are generated from imagery of the Context Camera (CTX) onboard the Mars Reconnaissance Orbiter (MRO) and are validated for clear and opaque atmospheric conditions. We analyze the necessity of using atmospheric compensation depending on the atmospheric conditions. For low optical depths, the Hapke model without an atmospheric component is still applicable to the Martian surface. For higher optical depths, atmospheric compensation is required to obtain good quality DEMs.

## 1. Introduction

The topography of planetary surfaces provides essential information for a wide field of applications. For example, geomorphologic analysis requires high-resolution Digital Elevation Models (DEMs), but the analysis of hyper-spectral data also benefits greatly from accurate DEMs. To correct hyper-spectral data for photometric effects and thermal emission, a detailed DEM and, in particular, accurate slopes are vital. Furthermore, the planning of rover missions relies on DEMs to assess landing sites and possible hazards associated with steep terrain. In recent years the interest in rover missions to Mars, in particular, has again increased significantly. In 2021, both the National Aeronautics and Space Agency’s (NASA’s) Perseverance Rover and the Chinese National Space Agency’s (CNSA’s) Zhurong rover successfully landed on the Martian surface. The Rosalind Franklin rover of the European Space Agency (ESA) was planned to be launched to Mars in 2022, but has been postponed to a later date. The Martian surface is covered by a thin and non-negligible atmosphere that increases the model complexity of intensity-based reconstruction methods. This study contributes in two ways. First, it adopts the previous framework of Grumpe and Wöhler [1], which was developed for Lunar DEM generation, and tailors it to Martian conditions. Therefore, the Hapke reflectance model is combined with an atmospheric model, yielding a state-of-the-art Shape from Shading (SfS) procedure. The relevant parameters can either be completely retrieved from the image or supplied from independent atmospheric measurements. Secondly, we investigate the performance of the procedure depending on the atmospheric conditions and parameter choices. This step has largely been neglected by previous studies on intensity-based Martian 3D-reconstruction and is crucial to evaluate the performance of SfS on Mars.

### 1.1. DEM Generation for Planetary Surfaces

In general, there are three major methodological approaches that are used to generate DEMs of planetary surfaces, i.e., ranging techniques, photogrammetric approaches and shading-based methods. Laser-altimetry samples the surface with laser pulses and the time-of-flight of the photons is translated into range measurements. The resulting DEMs have a high vertical fidelity and an extensive coverage of the planet is achieved. However, the lateral resolution is limited due to a comparatively coarse sampling of the surface (e.g., LOLA: 59 m/pix [2], MOLA: 463 m/pix [3]). Photogrammetric approaches, or stereo vision, use two or more images taken from different perspectives to infer a DEM, usually based on bundle adjustment. These methods do not require any physical reflectance model and are known to yield a good absolute height estimate. The state-of-the-art frameworks commonly employed in the planetary community are the Ames Stereo Pipeline [4] and BAE Systems’ SOCET SET^®^ [5]. Both rely on blockmatching to obtain tie-points for the bundle-adjustment procedure. Extensive regions of the planets are covered by regolith, which naturally appears textureless. The lack of texture may cause mismatching, which yields a variety of reconstruction artifacts such as spikes, holes where no matches could be generated and stair-like structures termed pixel-locking [6]. These stereo artifacts effectively lower the resolution of the DEM to several times below the pixel resolution.

Shading-based methods require a reflectance model to connect radiance measurements and surface gradients. They also need proper initialization to ensure convergence. This additional effort is justified by obtaining a DEM of pixel level resolution with a very accurate reconstruction of slopes and heights and the elimination of stereo artifacts, especially in textureless areas (e.g., [7]). In order to enhance the quality of DEMs, recent approaches have successfully combined low resolution DEMs from photogrammetry and laser-altimetry with SfS, among others, on either the Moon (e.g., [1,8,9,10]), Mercury (e.g., [11]) or on Mars (e.g., [12,13,14,15,16,17,18]). The rationale is that the low resolution DEM provides a good absolute height estimate and SfS is used to refine the surface such that the whole procedure combines the advantages of both approaches.

Recently, various works have explored machine-learning techniques to directly infer a relationship between measured image intensities and surface height (e.g., [19,20,21]). These approaches are purely data-driven and do not incorporate physical information. However, the training procedures implicitly learn the atmospheric conditions present in the training data, but the atmospheric influence has not been explicitly investigated in these works. Consequently, machine-learning approaches will also benefit from investigating the influence of the atmosphere on the reconstruction.

### 1.2. Shape from Shading in Planetary Remote Sensing

Galileo Galilei stated that surfaces that are tilted away from the sun appear darker and parts that face the sun appear brighter [22].This is probably the first time that surface shading was used to analyze the topography of a planetary body, and it has been used ever since. With the rise of more rigorous physical approaches and an increase in computational power, numerous approaches have been developed that use illumination geometry and shading to quantify the surface slope and topography, primarily of the Moon. In planetary science, these techniques are often termed photoclinometry, and the earliest approaches are given by Rindfleisch [23], Wildey [24], and Kirk [25]. A highly recognized technique from the computer vison community is the SfS method of Horn [26], which allows for the integrated recovery of slopes and heights. The surface reconstruction problem is encoded in terms of variational calculus and hence it is solved by minimizing a functional that penalizes the deviations between the shaded surface and the input image. To ensure integrability of the estimated surface, an additional regularization term is introduced. Further constraints were introduced by Shao et al. [27] to elastically tie the surface to a low frequency constraint surface, which improves the absolute vertical fidelity. Grumpe and Wöhler [1] generalized the variational approach and introduced a formalism to concurrently estimate the surface heights and the local reflectance properties in terms of the Hapke reflectance model, i.e., the albedo. Other recent approaches, which share many structural similarities, are, for example Wu et al. [9], Jiang et al. [18], and Alexandrov and Beyer [10]. Wu et al. [9] and Alexandrov and Beyer [10] use LRO-NAC images of the Moon and make use of the Lunar-Lambert model. The latter method additionally allows for multiple images. Jiang et al. [18] adopt the algorithm of Grumpe and Wöhler [1] and use the Mars-specific reflectance model and the Mars ReCo algorithm from Ceamanos et al. [28] to estimate the reflectance and atmospheric parameters.

### 1.3. Shape from Shading Applied to Mars

SfS has been applied to the Martian surface for forty years, and over time, different approaches of an increasing level of sophistication have been presented. This section provides a brief review of previous approaches for SfS applied to Mars and points out the methodical challenges and open questions addressed by this paper.

Previous approaches primarily differ in the reflectance model, the specific implementation of the SfS algorithm and the dataset that determines the actual resolution. Early works applied photoclinometry to Mariner 9 images [24] and SfS to Viking imagery [12,13]. Dorrer et al. [29] and Dorrer et al. [14] employ SfS to refine stereo DEMs, which are derived from the High-Resolution Stereo Camera (HRSC) onboard the Mars Express orbiter [30]. O’Hara and Barnes [16] propose the Large Deformation Optimization Shape from Shading (LDO-SfS) technique for recovering the surface shape without initialization. All approaches use simple reflectance models such as Lambert or Oren–Nayar [31], and mostly assume a constant albedo, if any, and do not model any atmospheric effects (e.g., [16]). Gehrke [15] is the first to address atmospheric effects on Mars in the context of surface reconstruction. The work combines facet stereo vision with SfS, applied to HRSC imagery. The Lunar-Lambert model is used for radiometric modeling, and the optical depth of the atmosphere is estimated from two HRSC images acquired under different observation angles.

Jiang et al. [18] were the first to propose a scheme for an integrated stereo and SfS approach on CTX images (up to 5 m/pix) with atmospheric compensation. They employ their previous reflectance model [28] to build a thorough physical reflectance and atmospheric model based on additional multi-angle CRISM measurements to estimate the model parameters. Compared to the photogrammetric reconstruction, the DEM results show improvement and are consistent with a sparse set of MOLA points. In a subsequent publication, Douté et al. [32] extend their approach to work with HiRISE imagery; due to the lack of validation data, the Isotropic Undecimated Wavelet Transform (IUWT) is employed for a consistency-check.

Despite the overall viability and adequate results of SfS on Mars, we identify two methodological challenges and one open question related to atmospheric compensation. First, all previous works, and most recently the approach of Jiang et al. [18], assume constant albedo throughout the scene. If a more sophisticated area with locally varying albedo is examined, Jiang et al. [18] perform clustering and divide the scene into multiple regions with constant albedo. Even though algorithms for SfS with locally varying albedo exist (e.g., Wu et al. [9] and Grumpe and Wöhler [1]), they have not yet been fully explored for Mars. Secondly, modeling the atmosphere remains challenging and largely unexplored. Many studies have simply neglected atmospheric effects and Gehrke [15] only estimated the optical depth. The approach of Jiang et al. [18] requires external data to estimate the parameters of the reflectance model and the atmospheric model. Significant additional processing is required for obtaining the image parameters. Due to the lack of such data, Jiang et al. [18] assume globally averaged parameters. In general, it would be beneficial to directly estimate the atmospheric parameters from the image data such that no external information is necessary. Thirdly, Jiang et al. [18] employed an atmospheric model for their SfS implementation, but did not provide any further analysis of the influence of atmospheric conditions on the reconstruction results. In fact, they utilized images acquired under extremely clear atmospheric conditions with an optical depth of τ≈0.16 (G20_025904_2209_XN_40N102W), τ≈0.08 (B20_017600_1538_XN_26S183W), and τ≈0.19 (B21_017786_1746_XN_05S222W), as can be retrieved from the maps of Montabone et al. [33]. Hess et al. [7] combined an atmospheric model with the SfS procedure from Grumpe and Wöhler [1] and Grumpe et al. [8]. They provided a short comparison with and without an atmospheric model for the first scene from Jiang et al. [18] (G20_025904_2209_XN_40N102W). Even the model without atmospheric compensation produced good results. However, none of the previous works have analyzed the influence of atmospheric modeling on SfS results for varying atmospheric conditions. Therefore, it remains unclear whether the effort of atmospheric compensation is always necessary and how the SfS procedure performs under increasing atmospheric opacity.

This work addresses these methodical challenges and the open question. The atmospherically compensated shape and albedo from shading approach extending on Grumpe and Wöhler [1] is outlined. The method employs Bayesian optimization for atmospheric parameter estimation. Further, this work aims to investigate whether external atmospheric parameters from, e.g., Montabone et al. [33] or Montabone et al. [34] are necessary or if these parameters can be directly retrieved from the scene. In order to systematically analyze the influence of varying atmospheric conditions on the performance of Martian SfS, three images of the same region as in Hess et al. [7] and Jiang et al. [18], but with good (τ≈0.16), medium (τ≈0.61), and bad (τ≈0.94) atmospheric conditions [33,34] are used.

## 2. Methods

The core idea of Shape from Shading (SfS) is to use the shading information of a given surface to infer its shape or, more rigorously, a radiance image *I* is used to estimate the surface shape, which can be represented by a scalar potential *z*. The SfS reconstruction task comes with four requirements, which are (1) a radiance image *I*, (2) a task-specific reflectance model *R*, (3) a reconstruction algorithm and (4) a low-frequency initialization of the surface. For Mars, radiance images acquired by the Mars Reconnaissance Orbiter (MRO) serve as the shading input *I* (Section 2.1). Our newly devised Martian reflectance model *R* maps surface gradients to reflectance values and thus links the shape to the shading of the surface (Section 2.2). Further, location and observation time specific model parameters are estimated, which is crucial for the accurate description of the planet’s photometric behavior. The atmospheric parameters are retrieved in a one-shot estimation with Bayesian optimization. The surface reconstruction problem is essentially ill-posed because a surface’s gradient field with two components as well as the single-scattering albedo are estimated from a single radiance image using the SfS algorithm of Grumpe and Wöhler [1] (Section 2.3). The shape of the surface and the albedo are updated in each of the pyramid levels until the SfS in the final pyramid level is carried out on full resolution images. The regularization requires a stereo-derived DEM as a low frequency constraint that is also used to estimate the atmospheric parameters once before the hierarchical SfS scheme. The full procedure is outlined in Section 2.4.

### 2.1. Datasets

The Mars Reconnaissance Orbiter (MRO) [35] carries, among other sensors, two cameras that are suited for SfS due to their high resolution and the range of usual observation angles. The High Resolution Imaging Science Experiment (HiRISE) [36] narrow angle camera captures images of selected target sites with a resolution of 25 cm/pix at best. The Context Camera (CTX) [37] contextualizes HiRISE imagery [36] at 5 m/pix resolution at best. CTX imagery covers the whole planet and many sites have been mapped by multiple images such that stereo reconstruction is possible. HiRISE only sparsely maps the planet and, often, there is no available stereo pair. Before processing, the raw data are calibrated to the reflectance factor I/F with ISIS3 [38]. The initial stereo DEMs and the HiRISE DEM for validation are calculated using the Ames stereo pipeline [4]. Additionally, the dust optical depth maps of Montabone et al. [33] and Montabone et al. [34], which are based mainly on data from the Mars climate sounder also onboard the Mars Reconnaissance Orbiter, can be used.

### 2.2. Reflectance Model

The reflectance model links the surface geometry to the radiance acquired by the sensor and, hence, serves as the crucial element of the SfS method. To model the reflectance properties of planet Mars, a photometric model for planetary surfaces (Section 2.2.1) is combined with an atmospheric model (Section 2.2.2).

#### 2.2.1. Photometric Models for Planetary Surfaces

Shepard [39] gives an overview of photometric models in planetary remote sensing. The simplest but crudest approximation is to assume an isotropic surface and apply Lambert’s reflectance model, as done by Dorrer and Zhou [13] and Hartt and Carlotto [12].

The empirical Lunar-Lambert model is a weighted superposition of Lambert’s law and the Lommel–Seeliger law with an additional phase function to address anisotropy, and was originally tailored to the Lunar surface [40]. Wu et al. [9] and Alexandrov and Beyer [10] use the Lunar-Lambert model for the Moon and Lohse et al. [41] and Gehrke [15] adopt it to Mars. The models of Shkuratov et al. [42] and Hapke (summarized in Hapke [43]) rely on a more rigorous derivation. They are advantageous since they take physical properties into account and are proven to work well for particulate materials. The Hapke model, especially, has sound quantitative support and can be regarded as the standard model for planetary photometry. Nevertheless, some shortcomings are discussed by Shepard and Helfenstein [44] and Shkuratov et al. [45].

In the earth remote sensing community, the semi-empirical kernel method is commonly employed to approximate the bi-directional distribution function (BRDF) of a surface by a weighted superposition of kernels [46]. These kernels are chosen from a catalog according to the scene type under investigation. Jiang et al. [18] combine Ross-Thick and Li-Sparse (RTLS) kernels for Mars as described by Ceamanos et al. [28]. The performance of this method was compared to the Hapke model, showing a good agreement in common cases, but stronger deviations, especially under large incidence angles [28].

This approach relies on the Hapke model as the de-facto standard of photometric planetary analysis, which has successfully been employed for SfS [1,8]. Several formulations of the Hapke model exist. The Anisotropic Multiple Scattering Approximation (AMSA) of the Hapke model is given by:

(1) $$r_{\mathrm{AMSA}}\left(\mu_{0},\mu ,g\right)=\frac{w}{4\pi }\frac{\mu_{0}}{\mu_{0}+\mu }\left\{p\left(g,\lambda \right)B_{\mathrm{SH}}\left(g\right)+M\left(\mu_{0},\mu \right)\right\}B_{\mathrm{CB}}\left(g\right).$$

The model is a function of a variety of photometric and geometric parameters. The main parameter is the single-scattering albedo *w*. Additionally, the opposition effect is modeled with the terms $B_{SH}\left(g\right)$ denoting the Shadow Hiding Opposition Effect (SHOE), and $B_{CB}\left(g\right)$, for the Coherent Backscatter Opposition Effect (CBOE). Both terms depend on two parameters each: one parameter for the strength of the opposition surge ($B_{S0}$/$B_{C0}$) and another parameter that describes the shape or width of the effect ($h_{s}$/$h_{c}$). In the model, multiple scattering within the medium is accounted for by the approximation $M(\mu_{0},\mu )$, which is given by:

(2) $$M\left(\mu_{0},\mu \right)=L_{1}\left(\mu_{0}\right)\left[H\left(\mu \right)-1\right]+L_{1}\left(\mu \right)\left[H\left(\mu_{0}\right)-1\right]+L_{2}\left[H\left(\mu \right)-1\right]\left[H\left(\mu_{0}\right)-1\right].$$

The geometry is defined by the cosines of the incidence and the emission angle $\mu_{0}=cos\left(i\right)$ and μ=cos(e), respectively. The angle *g* is termed the phase angle and is measured between the incoming light direction and the direction of the emitted light. The material dependency is addressed by the phase function p(g) and the single-scattering albedo *w*. The phase function can be expressed in terms of Legendre polynomials with the material specific Legendre coefficients $b_{n}$:

(3) $$p\left(g,\lambda \right)=1+\sum_{n=1}^{\infty }b_{n}\left(\lambda \right)P_{n}\left(\cos\left(g\right)\right).$$

Alternatively, the double-Henyey–Greenstein function can be used to approximate the phase function with only two material-specific parameters *b* and *c*:

(4) $$p\left(g\right)=\frac{1+c}{2}\frac{1-b^{2}}{{\left(1-2b\cos\left(g\right)+b^{2}\right)}^{3/2}}+\frac{1-c}{2}\frac{1-b^{2}}{{\left(1+2b\cos\left(g\right)+b^{2}\right)}^{3/2}}.$$

The Ambartsumian–Chandrasekhar *H*-function is defined as

(5) $$H\left(x\right)=1+\frac{w}{2}xH\left(x\right)\int_{0}^{1}\frac{x^{\prime }}{x+x^{\prime }}dx^{\prime }.$$

It is not analytically solvable, but viable approximations [43] exist:

(6) $$H\left(x\right)={\left\{1-wx\left[r_{0}+\frac{1-2r_{0}x}{2}\ln\frac{1+x}{x}\right]\right\}}^{-1}$$

with

(7) $$\begin{array}{cc} \gamma & =\sqrt{1-w} \end{array}$$

(8) $$\begin{array}{cc} r_{0} & =\frac{1-\gamma }{1+\gamma }. \end{array}$$

The remaining terms for $L_{1}$ and $L_{2}$ are defined by:

(9) $$\begin{array}{cc} L_{1}\left(\mu \right) & =1+\sum_{n=1}^{\infty }A_{n}b_{n}P_{n}\left(\mu \right), \end{array}$$

(10) $$\begin{array}{cc} L_{1}\left(\mu_{0}\right) & =1+\sum_{n=1}^{\infty }A_{n}b_{n}P_{n}\left(\mu_{0}\right), \end{array}$$

(11) $$\begin{array}{cc} L_{2} & =1+\sum_{n=1}^{\infty }A_{n}^{2}b_{n}. \end{array}$$

The coefficient $A_{n}$ is calculated by evaluating the following definition

(12) $$\begin{array}{cc} A_{0} & =0 \end{array}$$

(13) $$\begin{array}{cc} A_{n} & =\frac{{\left(-1\right)}^{(n+1)/2}}{n}\frac{1\;\cdot \;3\;\cdot \;5\cdots n}{1\;\cdot \;2\;\cdot \;4\cdots (n+10)},n\in \left\{2k|k\in N^{+}\right\}. \end{array}$$

#### 2.2.2. Atmospheric Model

Mars is encircled by a thin, but non-negligible atmosphere, which alters the radiance emerging from the planet’s surface. The mean surface pressure on Mars is approximately 6.3 hPa, but is highly variable compared to Earth [47]. The atmosphere consists almost entirely of CO_2_ (≈95%) [47], but the largest effect on the optical opacity of the atmosphere is caused by finely grained dust (effective radius approximately 1.4–1.7 μm [48]). Especially during the latter part of the Martian year, winds faster than 30 m/s [49] can raise large amounts of dust, leading to a higher optical depth. The effects that arise from the presence of an atmosphere are addressed by embedding the reflectance model into an atmospheric model. The atmospheric model, which serves the purpose of accurate surface estimation, must be physically plausible and accurate, while staying at a manageable level of complexity. These requirements can be balanced by making a set of assumptions, which can roughly be grouped into either arising from the geometry of the atmospheric model or from the physical effects that govern the interaction between light and the atmosphere.

**Figure 1:**
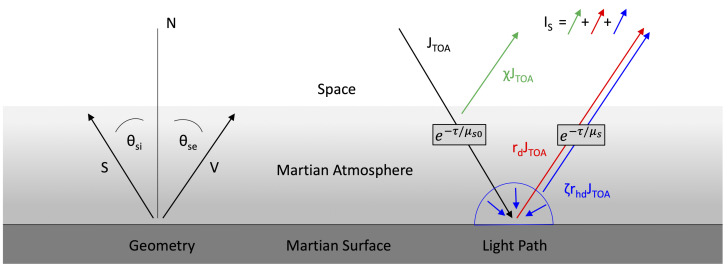
Scheme of the atmospheric model. **Left**: geometric definitions. **Right**: atmospheric effects.

The Martian atmosphere can be modeled as a horizontally stratified layer on top of a flat planetary surface. The geometrical constraints for our atmospheric model are accompanied with three simplifying assumptions. (1) Because the region of interest is rather small compared to its distance to the orbiter and to the sun, we omit small angular changes and assume constant orbiter zenith angles and constant solar zenith angles. (2) Further, the topographical changes within the region of interest are small compared to the thickness of the atmosphere, which allows us to neglect them as well. (3) Unlike Earth, Mars usually does not exhibit strong weather changes on local scales and hence we can assume constant atmospheric parameters for our region of interest. If cases arise in which local weather events are present or very strong topographic changes occur, they may be addressed individually by dividing the region of interest into smaller segments. A sketch of the atmospheric slab model can be found in Figure 1.

The total radiation that arrives at the image sensor is, in principle, a superposition of reflected and scattered radiation and thermally emitted radiation. Because the spectral ranges of CTX (500–700 nm [37]) and HiRISE (400–1000 nm, more specifically, 550–850 nm for the red channel [36], exclusively used in this work) imagery are in the visible region, thermal contributions in the infrared range are not relevant. The reflected and scattered light of a planet $I_{\mathrm{sensor}}$ which arrives at the sensor is commonly modeled [50] as the superposition of three contributing processes, i.e., directly reflected light $I_{\mathrm{direct}}$, reflected skylight $I_{\mathrm{sky}}$ and path-scattered light $I_{\mathrm{path}}$

(14) $$I_{\mathrm{sensor}}=I_{\mathrm{direct}}+I_{\mathrm{sky}}+I_{\mathrm{path}}.$$

The processes that govern the contributions are sketched in Figure 1 (right) and are outlined in the following paragraphs.

**Directly Reflected**

The Lambert–Beer law [51,52] states that the incoming light $J_{\mathrm{TOA}}$, which travels along a path *S* through the atmosphere with extinction E(s), is attenuated exponentially. If the optical depth τ of the atmosphere is defined as the line integral of the extinction E evaluated along a line *N* normal to the surface, we need to divide by the cosine of the solar zenith angle $\mu_{s}0$ to project it to the path of oblique illumination *S*. The top-of-atmosphere irradiance is denoted by $J_{\mathrm{TOA}}$ and the irradiance that reaches the Martian surface $J_{\mathrm{GND}}$ is given by

(15) $$J_{\mathrm{GND}}=J_{\mathrm{TOA}}e^{-\int_{S}E\left(s\right)ds}=J_{\mathrm{TOA}}e^{-\frac{\int_{N}E\left(z\right)dz}{\mu_{s}0}}=J_{\mathrm{TOA}}e^{-\frac{\tau }{\mu_{s}0}}.$$

The radiance $J_{\mathrm{GND}}$ is reflected by the Martian surface and travels back to the orbiter’s image sensor along the line *V* under the orbiter zenith angle $\theta_{\mathrm{se}}$. The cosine of this angle is denoted as $\mu_{s}$. En route, the light is attenuated a second time, such that $I_{\mathrm{direct}}$ becomes

(16) $$I_{\mathrm{direct}}=J_{\mathrm{TOA}}e^{-\tau \left(\frac{1}{\mu_{s0}}+\frac{1}{\mu_{s}}\right)}r\left(\mu_{0},\mu ,g\right).$$

**Skylight**

Parts of the incoming radiance $J_{\mathrm{TOA}}$ are scattered by the atmosphere and act as a diffuse source of illumination, termed skylight. The skylight $J_{\mathrm{TOA}}\zeta$ illuminates the Martian surface diffusely and is reflected into the direction of the sensor. This process can be directly modeled by employing the hemispherical-directional reflectance $r_{\mathrm{hd}}$, which links diffuse illumination to a collimated detector:

(17) $$I_{\mathrm{sky}}=J_{\mathrm{TOA}}\zeta r_{\mathrm{hd}}\left(\theta_{i},\theta_{e},g\right)e^{-\frac{\tau }{\mu_{s}}}.$$

The factor ζ describes the fraction of light that is actually scattered by the atmosphere to serve as diffuse skylight. The factor is obtained empirically in the parameter estimation step of our method. Following Nicodemus [53] and Hapke [43], the hemispherical-directional reflectance can be obtained by integrating the bi-directional reflectance *r* over the upper half sphere:

(18) $$r_{\mathrm{hd}}\left(\theta_{e}\right)=\int_{\theta_{e}}^{\pi /2}\int_{\phi }^{\pi /2}r\left(\theta_{i},\theta_{e},g\right)\mu \sin\left(\theta_{i}\right)d\phi d\theta_{i}.$$

The bi-directional reflectance *r* can be implemented by Hapke’s model (Equation (1)). It is feasible to assume that the narrow peaks of the opposition effects have a neglectable contribution to the whole integral over the upper half sphere. Therefore, we can set $B_{\mathrm{SH}}=1$ and $B_{\mathrm{CB}}=1$ and arrive at a simpler formulation of Hapke’s model. The integral then evaluates to

(19) $$\begin{array}{cc} r_{\mathrm{hd}}\left(\mu \right)= & 1-\gamma H\left(\mu \right)+\sum_{n=1}^{\infty }b_{n}\left\{P_{n}\left(\mu \right)+A_{n}\left[H(\mu )-1\right]\right\}\\ \; & \times \{\frac{w}{2}\sum_{k=0}^{\left\lfloor n/2\right\rfloor }{\left(-1\right)}^{k}\frac{(2n-2k)!}{\left(n-k\right)!\left(n-2k\right)!k!2^{n}}\\ \; & \;\cdot \;[\mu^{n-2k+1}\left(\left(log\left(\mu \right)-log\left(\mu +1\right)\right)\;\cdot \;{\left(-1\right)}^{n-2k}\right)\\ & +\sum_{j=0}^{n-2k}\frac{{\left(-\mu \right)}^{j}}{n-2k+1-j}]\\ \; & +A_{n}\left[\frac{1}{H(\mu )}-\gamma \right]-A_{n}\frac{w}{2}\left[1-\mu \left(ln\frac{1+\mu }{\mu }\right)\right]\}. \end{array}$$

**Path-Scattered**

Finally, some parts of the light are scattered by the atmosphere into the direction of the sensor due to *“Rayleigh scattering and aerosol and particulate Mie scattering and [it] is reasonably assumed to be constant over the entire scene”* [50]. Thus, we state

(20) $$I_{\mathrm{path}}=\chi J_{\mathrm{TOA}}.$$

#### 2.2.3. Full Model and Parameter Estimation

Summarizing the components (Equations (16), (17) and (20)) yields the full atmospheric model:

(21) $$\begin{array}{cc} r_{M} & =\frac{I_{\mathrm{sensor}}}{J_{\mathrm{TOA}}}\\ \; & =\left(e^{-\frac{\tau }{\mu_{s}0}}r_{d}\left(\theta_{i},\theta_{e},g,w\right)+\zeta r_{\mathrm{hd}}\left(\theta_{i},\theta_{e},g,w\right)\right)e^{-\frac{\tau }{\mu_{s}}}+\chi . \end{array}$$

The full reflectance model $r_{M}$ depends on 16 parameters summarized in Table 1, which can be grouped into three distinct sets, i.e., geometric, atmospheric and material parameters. To accurately perform SfS, it is crucial to correctly obtain the reflectance parameters for a given scene and avoid interdependencies between parameters. The parameter determination is done in three ways. One set of parameters is given by a priori knowledge and, therefore does not need to be estimated. The second set of parameters is determined using a one-shot estimation based on the initial DEM and the image (see Section 2.2.4 for details). These are the atmospheric parameters in particular. The remaining geometric and material parameters are iteratively updated throughout the SfS method.

**Geometric**

The geometric parameters $\mu_{s}0$ and $\mu_{s}$ describe the cosine of the sun zenith angle and the orbiter’s zenith angle. Given the small angle approximation, they can be assumed to be constant over the entire image and are inferred from the ancillary data of the CTX or HiRISE imagery. The geometric parameters μ, $\mu_{0}$ and *g* are derived from the illumination vector, the observation vector and the normal vector of a pixel of the DEM. All three parameters require a DEM, but the parameters are also the input for the reflectance model, which is actually used to estimate the DEM. Due to this coupling, the geometric parameters μ, $\mu_{0}$ and *g*, as well as the albedo *w*, are estimated during the iterative shape and albedo from shading procedure (Section 2.3).

**Material**

The single-scattering albedo *w* is the most important material parameter and describes the ratio of reflected and incoming light that interacts with a single particle. It is generally assumed that the albedo varies locally, and hence it is estimated pixel-wise within the iterative shape and albedo from shading procedure. The initial albedo $w_{0}$ is assumed to be constant over the entire image and estimated once before the SfS procedure. Johnson et al. [54] retrieved Hapke’s single-scattering albedo *w* for different surface materials from pancam measurements of the Opportunity rover. In the red wavelength range for the CTX camera (500–700 nm) and the HiRISE camera (550–850 nm), the albedo is in the interval 0.35≤w≤0.85. Laboratory measurements of Martian dust analogs (HWMK919) even yield albedos of up to w=0.95 in the crucial wavelength range [55]. Thus, physically plausible albedo values on Mars are somewhere in the broad interval of 0.35≤w≤0.95. The phase function p(g) describes the phase-angle dependent scattering behavior of particles and can be approximated by the double-lobed Henyey–Greenstein function. This function depends on the backscattering parameter *b* and the asymmetry parameter *c*, which are related by the so-called hockeystick relation [56]. The parameters *b* and *c* are located on this curve depending on the material. However, as done in previous work (e.g., [1]), there is a working assumption to equip the phase function with constant parameters. For the Moon, we used globally averaged b=0.21 and c=0.7 from Warell [57]. For Mars, Fernando et al. [58] set b=0.3 and 0.6≤c≤1.0 for various materials observed by the pancam of Opportunity and Spirit. Johnson et al. [54] found slightly smaller values: c≈0.5. A globally averaged phase function based on CRISM and OMEGA observations was determined by Vincendon [59], who found b=0.12 and c=0.6. Given these data, we set c=0.6 and adopt b=0.12. Fernando et al. [58] also determined the roughness for various Martian materials, as seen by Spirit’s pancam. The unweighted average roughness of these materials is $\bar{\theta }=14.4^{\circ }$. Vincendon [59] derived a global roughness of $\bar{\theta }=17^{\circ }$. For this study, we omit the roughness to be able to compare the model with and without atmospheric compensation, because the roughness is not considered in the hemispherical-directional reflectance.

**Atmospheric**

The optical depth τ and the weights ζ for the diffuse illumination and χ for the path scattered radiance are assumed to be constant over the entire image and the estimation is performed once before the SfS algorithm is carried out. The optical depth of the Martian atmosphere undergoes seasonal variations and is usually in the range of 0.3≤τ≤0.7 [60]. The pancam of Opportunity rover reported values in the range of 0.43≤τ≤0.53 at its exploration site [60] and the 880 nm channel of the Spirit rover camera encountered values around τ=0.3 [61] for roughly a quarter of the Martian year. During the dust storm season later in the year, the optical depth rises well above τ≥1 and may reach levels up to τ≥3 [61], such that the atmosphere becomes opaque. The region of interest investigated in this work is at a higher latitude than the rovers, and the optical depth is usually lower compared to the equator [33]. For a reasonable reconstruction, we set 0.1≤τ≤3 as boundaries. The weight ζ is approximately the relative importance of the hemispherical reflected light compared to the directly reflected radiance. We do not expect the hemispherical reflected light to be stronger than 20% of the directly reflected light. Consequently, we set ζ to be between 0 and 0.2. In contrast to ζ, χ is an additive term. In the case of a global dust storm, almost all received radiance can be attributed to path scattered radiance—the surface is invisible. In most cases, however, it should only be a minor factor compared to the total radiance, which is in the order of $3\times 10^{-2}$ to $6\times 10^{-2}$. Therefore, we expect χ to be smaller than $2\times 10^{-2}$.

#### 2.2.4. One-Shot Parameter Estimation

The atmospheric parameters and the initial mean albedo $w_{0}$ are assumed to be constant over the scene and are estimated once before the SfS method and are not updated in the SfS scheme. Then, the parameters are adjusted such that the rendered image matches the measured I/F data from the CTX or HiRISE instrument. These atmospheric parameters that best reconstruct the image data are used throughout the SfS. The resulting albedo serves as a global initialization, which is then refined locally within the SfS algorithm. Some challenges were encountered when devising a scheme for parameter retrieval and, as follows, the solutions to these challenges form the final scheme:

Firstly, pixel-locking is a stereo artifact that yields stair-like structures at slopes, e.g., at crater walls. Consequently, the gradient of these areas oscillates between a value that is either too steep or too flat compared to the real slope. The surface reflectance model depends on the surface inclination towards the sun, which is derived from the gradients of the DEM. If the reflectance takes oscillating gradients, it predicts an oscillating reflectance, which is not observed on the planet. To mitigate this effect, a Gaussian filter with a standard deviation of 2 is applied to the initial DEM in MATLAB^®^ such that the stairs are smoothed out and the gradient becomes oscillation-free.

Secondly, the problem is ill-posed. Changes in the observed I/F value can originate from atmospheric effects, albedo changes or changes in the surface slope. For example, choosing a lower albedo or higher optical depth can darken the overall scene. The model relies on the assumption that the scene is described by constant atmospheric parameters and that the albedo changes are of lower spatial frequency. Most of the variations in brightness are explained by the shape of the surface. To disentangle these effects, it is best to use many different data points to cover the variations in the model. This is achieved by sampling areas with significant slope variations. Nonetheless, albedo changes can account for a change in I/F. To improve the parameter retrieval, it is best to sample areas with nearly constant albedo.

This also means that the parameters cannot be obtained entirely automatically. Experience and prior knowledge about the formation and the atmospheric conditions of the scene at time of acquisition are necessary. Physically reasonable boundaries for the parameters need to be chosen to constrain the optimization procedure. To address the given challenges, the following scheme for parameter retrieval is devised:

(1) The initial stereo DEM is filtered with a Gaussian filter to suppress pixel-locking;
(2) An I/F image is rendered from the smoothed, downsampled DEM using the combined atmospheric and reflectance model;
(3) Training profiles are sampled, which should:
  (a) not cross severe stereo artefacts such as spikes and holes,
  (b) cover enough variations of slopes (e.g., shadows, as well as bright facets oriented towards the sun),
  (c) have a constant albedo. This is seen if the image has brightness variations which are not correlated with slope variations;
(4) A Bayesian optimization algorithm in MATLAB^®^ (bayesopt) is used to impose constraints on the parameters and to avoid terminating in a local minimum (e.g., [62,63,64]).

### 2.3. Shape from Shading

SfS uses the shading of an object to infer its shape. It is commonly formulated as an optimization problem, but using one 2D image to estimate a 3D shape is essentially ill-posed (e.g., [25,26]). Including information from a lower resolution DEM can help constrain the procedure (e.g., [27]). The model includes three regularization terms [1], namely, the integrability error and the deviation of absolute heights [27] and gradients of the DEM from the initial DEM compared at a lower frequency. Choosing appropriate weights for these regularization terms is essential for the procedure to converge. The parameters are listed in Table 2. The values of $\tau_{SfS}$ and δ are chosen such that the weighted errors are of a similar order of magnitude as the intensity error. The weight for the integrability error is chosen such that it is the smallest value possible without the procedure to diverge. In this way, most details are reconstructed, and the resulting surface does not become too smooth. The technique is described by Grumpe and Wöhler [1] and Grumpe et al. [8]. A summary can be found in Appendix A.

### 2.4. Full Framework

**Figure 2:**
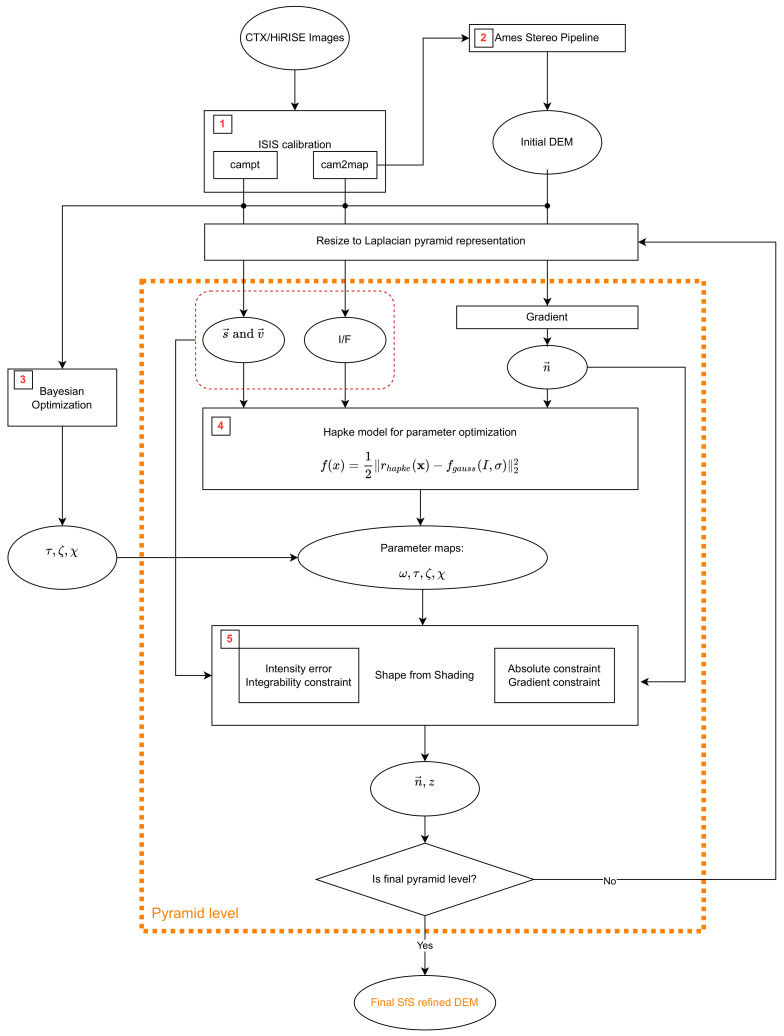
The complete SfS scheme. Steps in the orange dashed box are executed for each pyramid level at, e.g., 1/16, 1/8, 1/4, 1/2 and at full resolution.

The complete procedure is summarized in Figure 2. The initial data product from the CTX or HiRISE image pair is calibrated using ISIS3 ((1) in Figure 2). The images are calibrated to spectral radiance and both images of a pair are projected to the same simple-cylindrical map projection. With ISIS3, the orbiter and sun position can be retrieved with the campt command. These meta-data are used to calculate the vectors pointing to the orbiter ($\vec{v}$) and to the sun ($\vec{s}$) for each pixel. The radiance images are then used to generate the stereo DEM using the Ames stereo pipeline ((2) in Figure 2). Based on the low-pass filtered stereo DEM and the calibrated images, the atmospheric parameters (τ, ζ, χ) are estimated with Bayesian optimization ((3) in Figure 2). One ISIS3 calibrated radiance image and the initial DEM are reduced to a Laplacian pyramid representation. In this study, the procedure starts at the third pyramid level, which corresponds to 1/8th of the full resolution. In the next iteration, the resulting SfS DEM from the 3rd pyramid level is enlarged to 1/4th of the full resolution and is used as the initial DEM. The low frequency component of the albedo (Gaussian filter width σ) is estimated using the combined atmospheric and reflectance model ((4) in Figure 2) based on the surface gradients and the illumination and viewing conditions. The resulting albedo and the previously calculated atmospheric parameters are then used for the reflectance modeling in the SfS framework of Grumpe et al. [8] ((5) in Figure 2). The regularization constraints have to be weighted with hand chosen parameters. Depending on the choice of these regularization parameters the SfS algorithm converges faster or slower. If the regularization parameters are chosen poorly or the radiative transfer model is inadequate, the SfS might diverge. After convergence, the resulting DEM is then fed back into the procedure, serving as an initialization for the next higher pyramid level representation, except for the refinement at full image resolution. After applying SfS at the full resolution of the image, the refined DEM is the final result.

## 3. Results and Discussion

**Figure 3:**
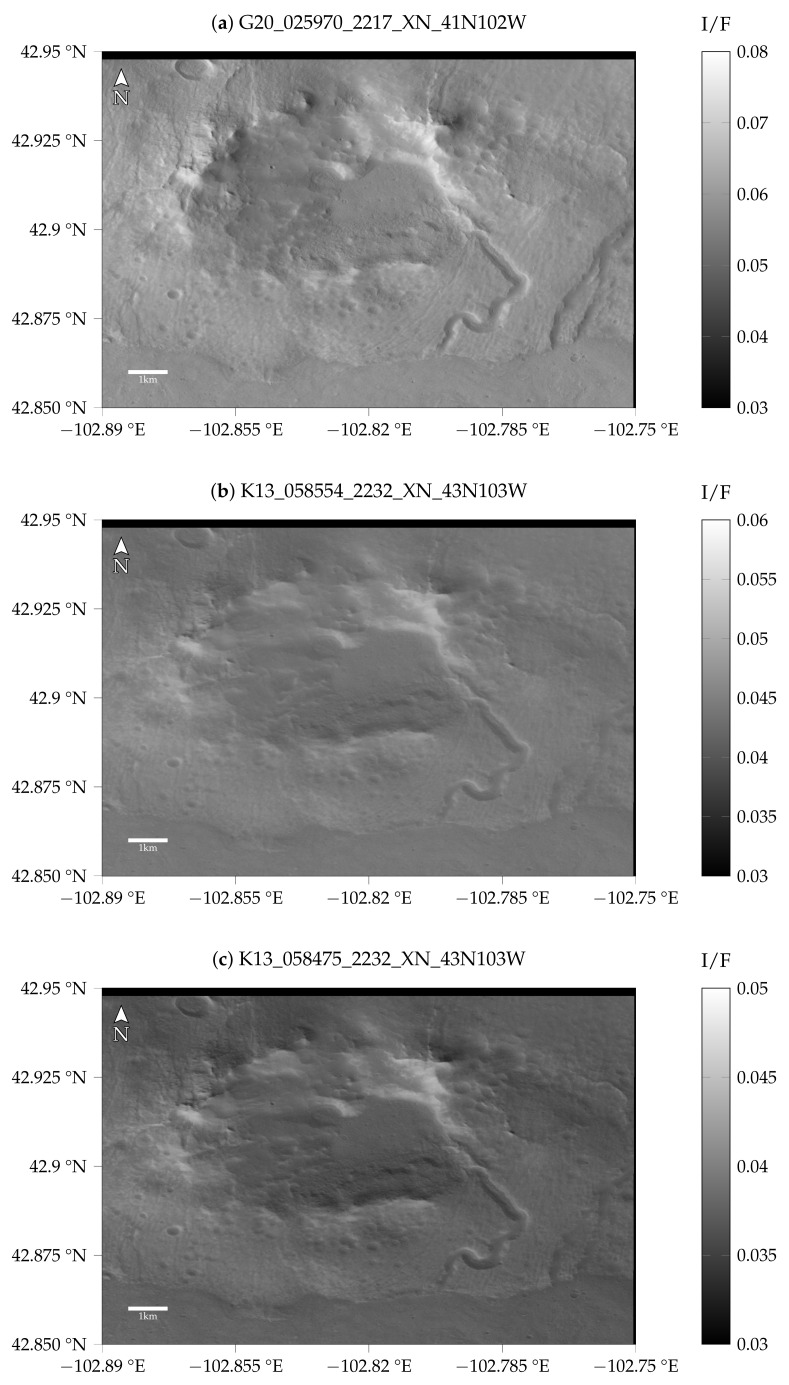
Images of the region of interest inside of the crater northeast of Alba Patera.

**Figure 4:**
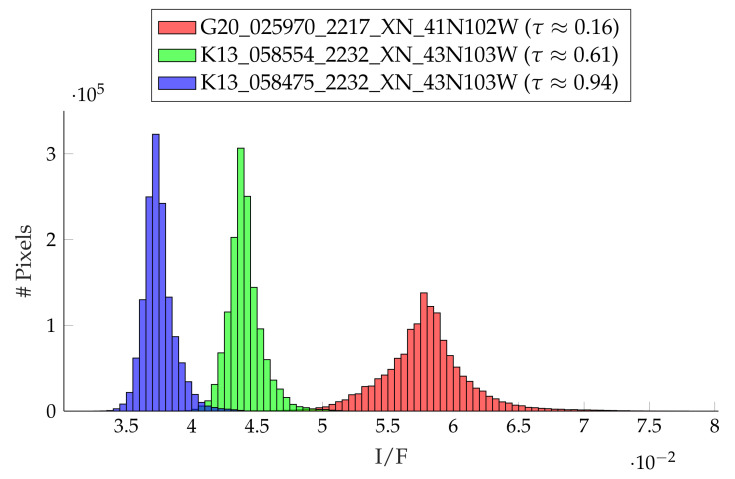
Radiance distributions for the three selected CTX images. The higher the optical depth becomes, the darker the images and the lower the contrast.

We carried out the following experiments to investigate the influence of the atmospheric conditions on the reconstruction procedure. We selected three CTX images for different atmospheric conditions listed in Table 3 for a region in the northeast of Alba Patera. The images were selected based on the corresponding dust optical depth (DOD) according to Montabone et al. [33] and Montabone et al. [34] at the region of interest at the time the images were taken. The DOD values from 9.3 μm were converted to the visible range by applying the factor 2.6 [33]. Therefore, these values can be seen as a general reference value, but are associated with significant uncertainties (see also, [33,34]). The images selected this way are displayed in Figure 3. It can be seen that the image for the good atmospheric conditions is significantly brighter compared to the other two images and shows higher contrast. This dichotomy is highlighted when plotting the histogram of the radiance values of the different images (see Figure 4). Visually, the difference between the CTX images K13_058554_2232_XN_43N103W and K13_058475_2232_XN_43N103W is small, but in the histogram, it can be seen that the image K13_058475_2232_XN_43N103W for τ=0.94 corresponds to a higher optical depth, meaning it is generally darker and exhibits lower contrast.

**Figure 5:**
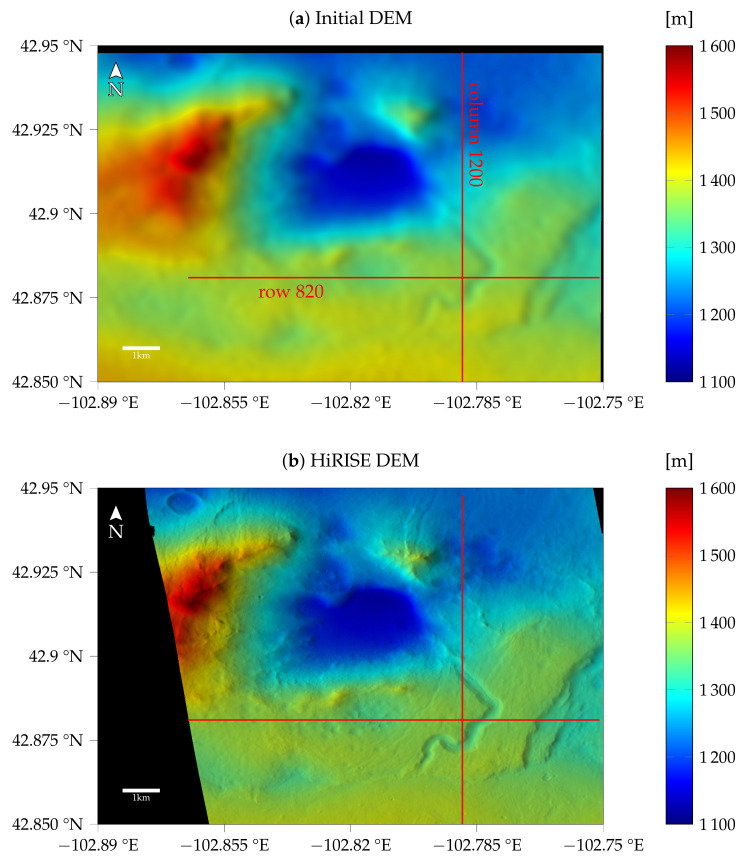
Initial DEM based on CTX stereo image pair and ground truth DEM based on HiRISE stereo image pair. Red lines indicate the profiles that will be investigated in this work.

We limited the region of interest to the area where a HiRISE stereo pair is available to obtain a DEM that can be considered a ground truth reference because of the higher resolution of the HiRISE images. The stereo DEMs are calculated using the Ames stereo pipeline [4]. The CTX stereo DEM that is used as an initialization at Laplacian pyramid level 3 is filtered with a Gaussian low pass filter (σ=2) to remove the strongest stereo artifacts and is shown in Figure 5. We used the initial DEM at full resolution, but filtered, to estimate the atmospheric parameters in Section 3.1. The HiRISE DEM is reduced to CTX resolution. Nonetheless, stereo artifacts are clearly visible in the shaded and color-coded DEM in Figure 5, especially at the slope in the west towards the lowest parts in the center and the north. Therefore, we selected profiles in the southern and eastern parts of the image, marked by the red lines in Figure 5. These profiles cover a variety of terrain, but are not affected as much by stereo artifacts. An offset is individually fitted for all different models and profiles to minimize the absolute deviation from the HiRISE ground truth.

The quality of the results is crucially dependent on the weight of the integrability error (γ), as described in Section 2.3. Therefore, we will analyze two values of γ, which we determine by using the heuristic described in Section 2.3 once for the AMSA model and once for the model with atmospheric compensation.

All images are co-registered to image G20_025970_2217_XN_41N102W, and for evaluation, all images are subsequently co-registered to the HiRISE stereo DEM. We used a piece-wise linear transformation instead of a projective transformation because the disparities due to the different perspectives seem to be smaller for the piece-wise linear transformation. The selected profiles for evaluation are also not strongly affected by possible differences in perspective. However, these differences would strongly influence the measured RMSE. Therefore, we limit our analysis to the selected profiles.

Thus, we define image G20_025970_2217_XN_41N102W to have *good atmospheric conditions* (see Section 3.2), image K13_058554_2232_XN_43N103W to have *medium atmospheric conditions* (see Section 3.3) and image K13_058475_2232_XN_43N103W to have *bad atmospheric conditions* (see Section 3.4). In Section 3.1, the atmospheric parameters are calculated once using the fixed optical depth from the maps of Montabone et al. [33] and Montabone et al. [34] and once with freely adaptable optical depth.

### 3.1. Parameter Estimation

**Figure A1:**
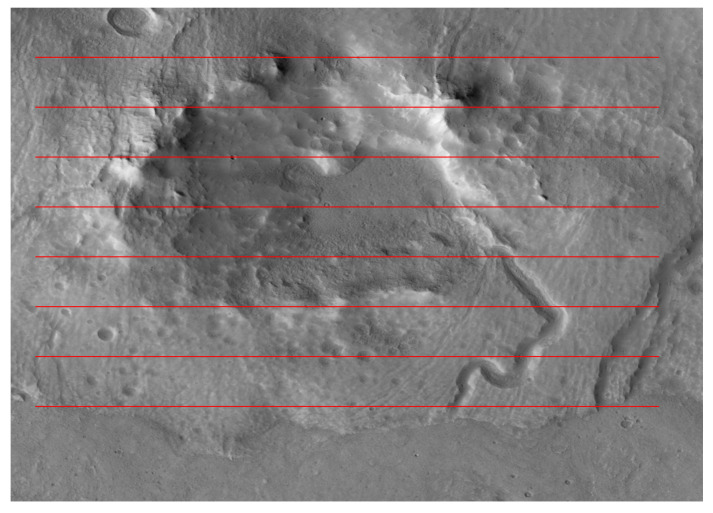
Profiles selected for the Bayesian optimization to determine the atmospheric parameters and the mean albedo.

The radiative transfer modeling relies on the reflectance model of Hapke [43] and the atmospheric model introduced in Section 2.2.2. For the atmospheric model (AMSA ATM), three parameters must be determined that are assumed to be constant over the image. For the three images, we carried out the procedure outlined in Section 2.2.4 using the profiles shown in Figure A1 (Appendix B).

**Figure 6:**
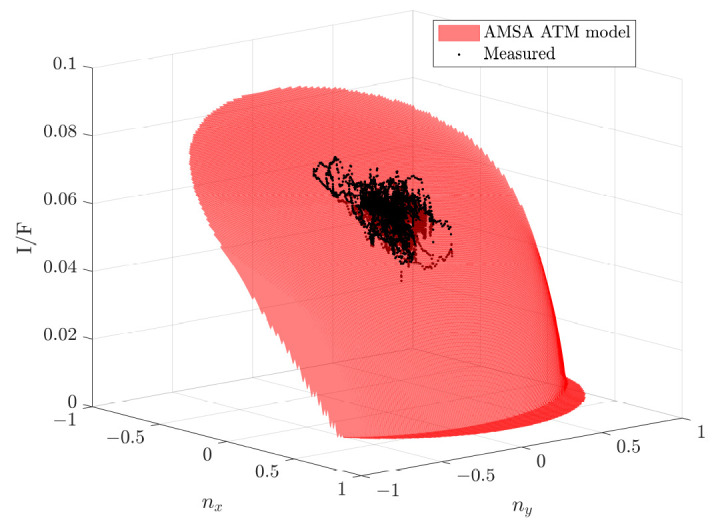
The estimated best fit model with atmospheric parameters is displayed in red and the measured I/F values for the normal vectors based on the initial DEM are represented by the black dots. The x-axis represents the x-component of the normalized normal vector and the y-axis represents the y-component of the normalized normal vector based on the initial DEM. Line patterns in the black point cloud are the result of stereo artifacts in the initial DEM.

The first image G20_025970_2217_XN_41N102W represents excellent atmospheric conditions and is thus considered a reference image for the parameter determination. We use the value for τ from Montabone et al. [33] to determine the other parameters, namely $w_{0}$, ζ and χ. The so determined mean single-scattering albedo is then used for the other images because the albedo should not change between the observations, and in order to remove mathematical dependencies between the optical depth and the single-scattering albedo. The resulting parameters are listed in Table 4. The fit between the model and the measured radiance values is displayed in Figure 6. The red surface represents the model for all possible normal vectors in the DEM. The x- and y-axes represent the x- and y-component of the surface normal vectors, respectively. The orientation of the surface is dependent on the illumination and viewing directions. However, all three images are taken under similar viewing and illumination conditions because the Mars Reconnaissance Orbiter’s orbit is sun-synchronous. The shape of the surface is defined by the parameters of the model. If τ, ζ and χ are equal to zero, the modeled radiance values above 90° incidence angle (shadows) becomes zero, making it equal to the Hapke model without atmospheric compensation. The larger ζ and χ become, the brighter the shadows become. The higher τ becomes, the darker the overall image becomes.

**Figure 7:**
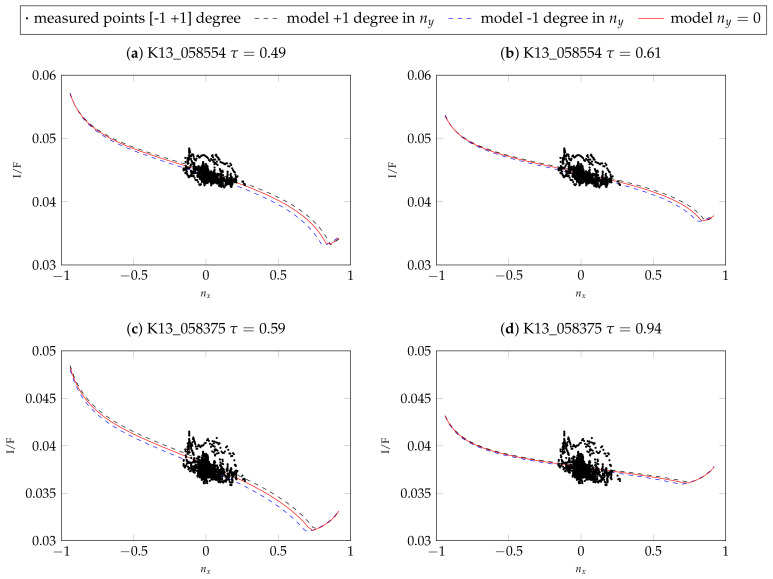
2D representations of the atmospheric model for the different parameters for the images K13_058554_2232_XN_43N103W and K13_058475_2232_XN_43N103W. The plots correspond to the planar intersection of the 3D representation (similar to Figure 6) and are created by omitting all points with a normal vector inclination of above 1° and below −1° in y-direction. The red line represents the model for $n_{y}=0$.

We reduced the 3D representation to a 2D representation for the other two images. Because of the observation geometry of the scene, the main variation of the model is along the x-component of the surface normal vector ($n_{x}$). Therefore, we plot I/F dependent on $n_{x}$, as seen in Figure 7. Because there is some dependence on y, for the 2D representation, we omit all measured values for which the surface normal vector has an inclination of above 1° and below −1° in y-direction. Furthermore, we plot the model for +1° and −1° inclination in the y-direction as the dashed lines.

The parameter values for the second image K13_058554_2232_XN_43N103W with medium atmospheric conditions are calculated once with a fixed value of τ=0.61 and once with a free optical depth. The respective best fit values for the parameters are listed in Table 4, and the modeled and measured radiance values as described in the previous paragraph are displayed in Figure 7. Because the overall brightness is lower in these images, the total RMSE is also generally smaller. Figure 7 shows that the two models are very similar, but for τ=0.61, the contrast is even further reduced compared to the free estimate of 0.49. Similarly, for the image K13_058475_2232_XN_43N103W, the estimated optical depth of 0.59 is smaller compared to the parameter from Montabone et al. [34] (τ=0.94). The quality of the fit for fixed τ appears to be inferior because the model becomes very flat. This is also visible in the RMSE of 0.00106 compared to the 0.00079 for the optimization with free τ (see Table 4).

### 3.2. Good Atmospheric Conditions

**Figure 8:**
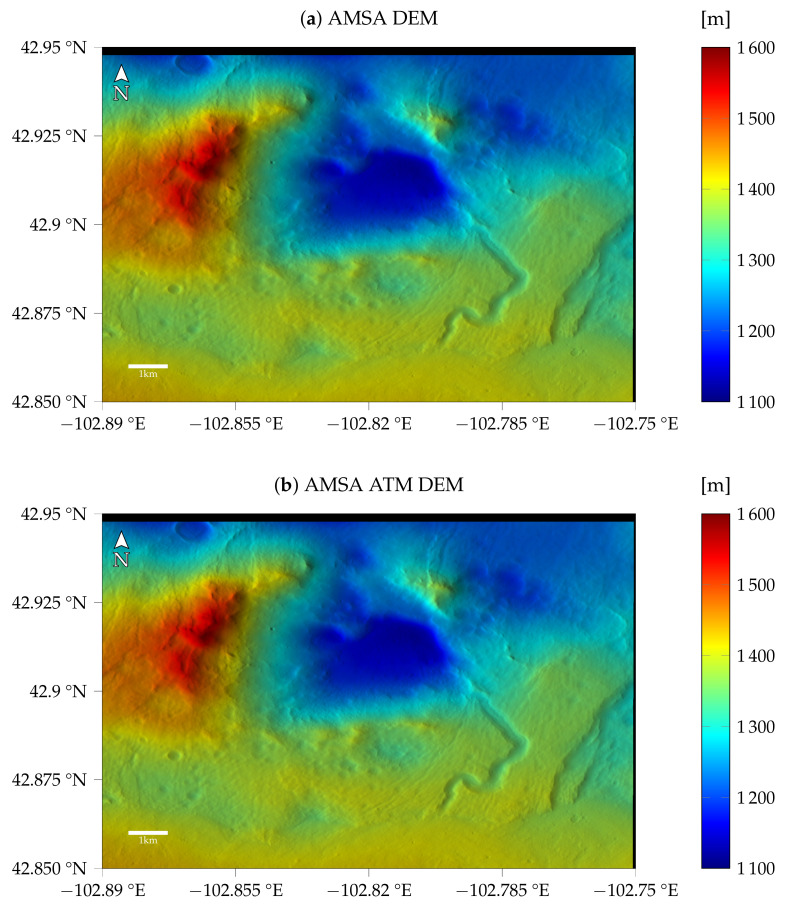
Results for image G20_025970_2217_XN_41N102W (**a**) without and (**b**) with atmospheric compensation. Here, no difference between the two DEMs is visible in the colorcoded shaded DEM.

For the first image G20_025970_2217_XN_41N102W, taken at a time with a low optical depth, the models with atmospheric compensation and the simple AMSA model are very similar, as can be seen in Figure 6, i.e., the shadows are estimated to be almost zero for the AMSA ATM model as well. The resulting DEMs for the same γ of $1\times 10^{-3}$ are shown in Figure 8. Qualitatively, no difference between the two color-coded DEMs can be observed. The shading with a fixed albedo overlaid on the color-coded DEM looks sharp and shows the small features that are also visible in the image. The visible quality of the DEM arguably exceeds the HiRISE stereo DEM, where stereo artifacts still pose a problem even when reduced to CTX resolution.

**Figure 9:**
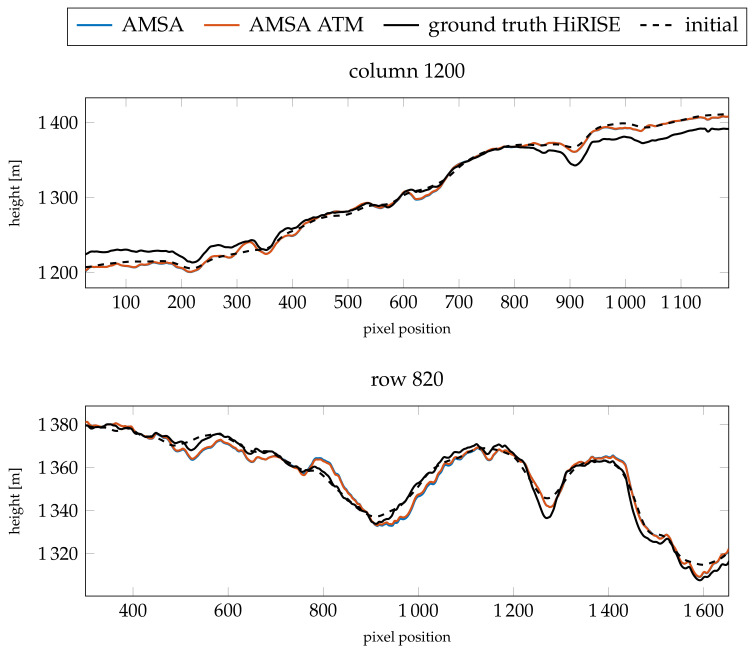
Resulting absolute heights compared for the two profiles investigated for image G20_025970_2217_XN_41N102W.

**Figure 10:**
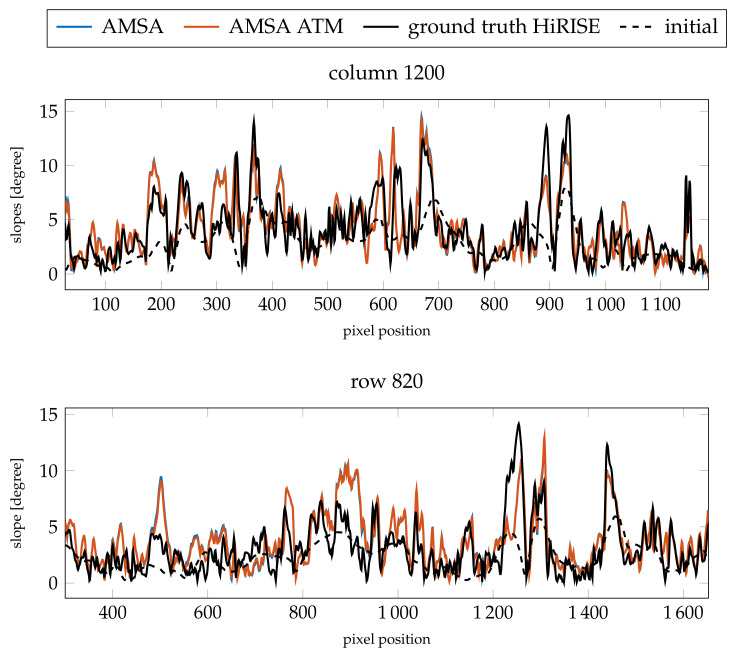
Resulting slopes compared for the two profiles investigated for image G20_025970_2217_XN_41N102W.

When investigating the profiles in Figure 9 and Figure 10 for heights and slopes, respectively, both are almost identical. Compared to the HiRISE ground truth, a similar level of detail is visible in the profiles, especially highlighted by the slopes. Some slopes, however, seem to be exaggerated by the SfS procedure. There appears to be a mismatch between the absolute heights of the ground truth and the initial DEM in column 1200. This mismatch is also visible in the absolute heights of the SfS results. The RMSE values are similar between the atmospheric and simple AMSA model (see Table 5), but the model with an atmospheric term consistently performs slightly better.

### 3.3. Medium Atmospheric Conditions

The second image represents medium atmospheric conditions. According to the maps of Montabone et al. [34], the optical depth corresponds to τ=0.61. We applied SfS for all combinations of $\gamma =3\times 10^{-3}$ or $\gamma =3\times 10^{-4}$ and for the model with atmospheric compensation τ=0.61 or τ=0.49. The value $\gamma =3\times 10^{-3}$ was determined by selecting the smallest value for which the AMSA model did not diverge and $\gamma =3\times 10^{-4}$, similarly, for the AMSA ATM model. The RMSE values for the examined profiles for all combinations are listed in Table 6.

For $\gamma =3\times 10^{-4}$, the simple AMSA model diverges almost entirely, such that the changes are discarded and the previous level Laplacian pyramid representation is carried over to the next higher pyramid level. Therefore, the error of the AMSA DEM is almost identical to the error of the initial DEM. Otherwise, both the model with an atmospheric compensation term and the simple AMSA model did not improve the absolute heights. The slopes improve for the model with an atmosphere for both values of γ in the case of column 1200, and only for $\gamma =3\times 10^{-4}$ in the case of row 820. For this image, the optical depth of 0.61 produces consistently better results compared to τ=0.49.

**Figure 11:**
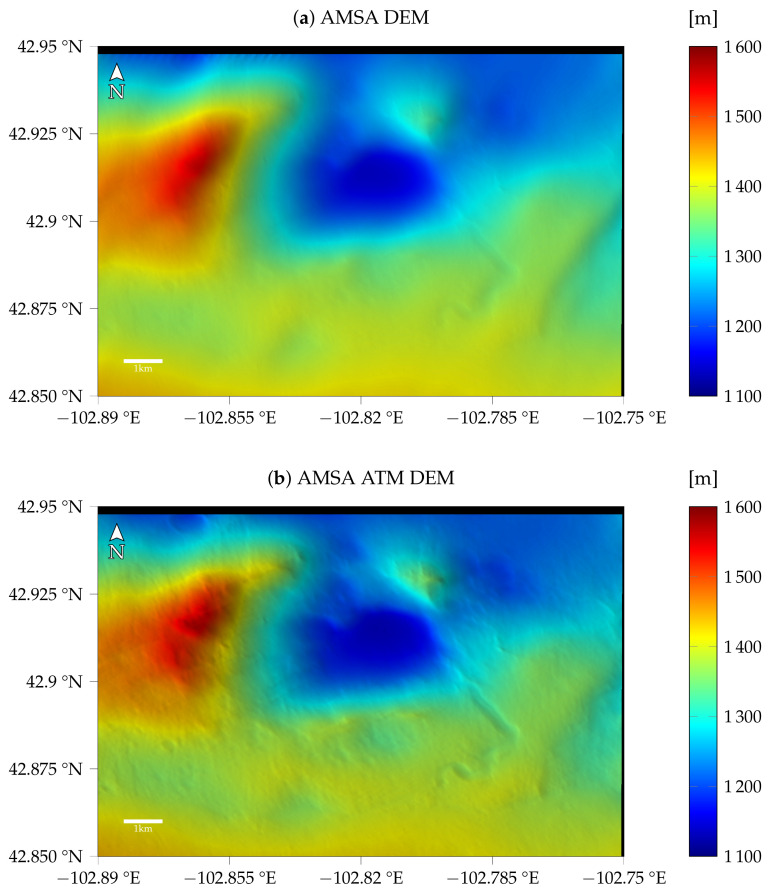
Results for image K13_058554_2232_XN_43N103W (**a**) without and (**b**) with atmospheric compensation. The results for $\gamma =3\times 10^{-3}$ and, in the case of the SfS with atmospheric compensation, τ=0.61 are shown.

**Figure A7:**
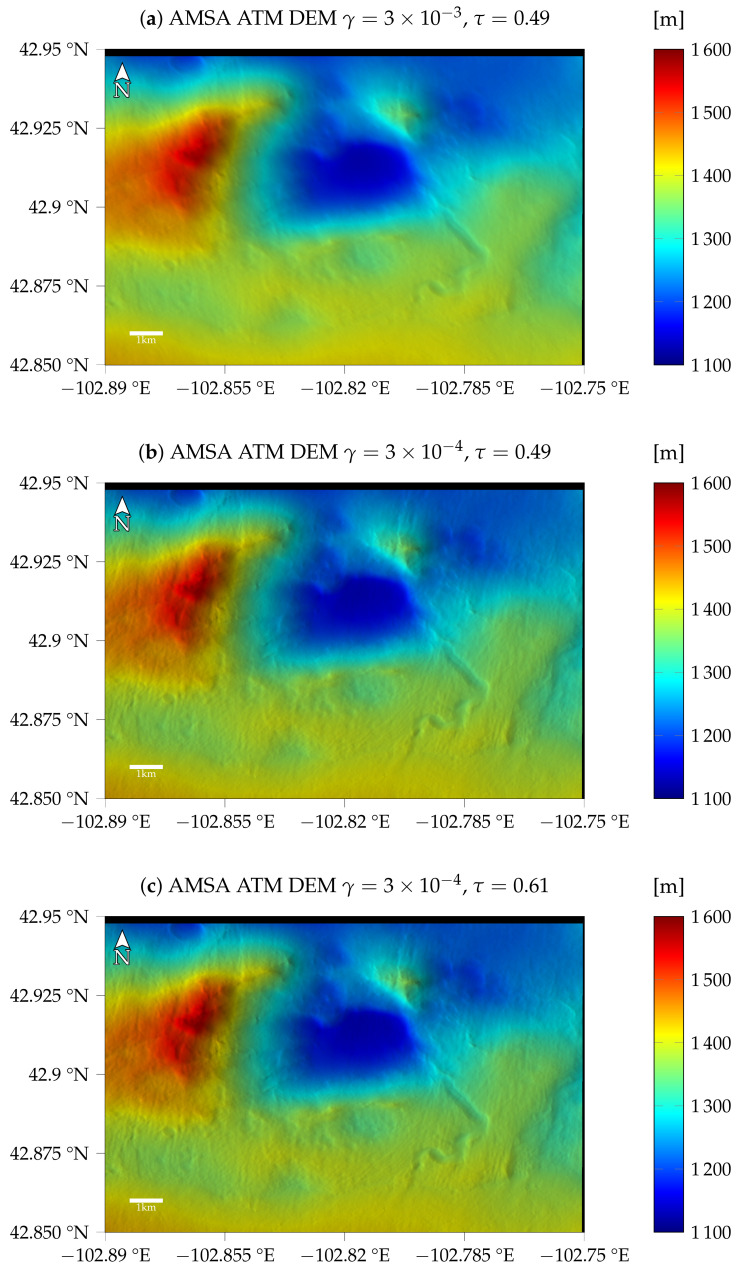
Color-coded and shaded DEMs created with the atmospherically compensated method not displayed in the main text of the manuscript for image K13_058554_2232_XN_43N103W (medium atmospheric conditions).

The resulting color-coded DEMs for $\gamma =3\times 10^{-3}$ and the fixed τ=0.61 are displayed in Figure 11. The AMSA DEM appears to be blurred compared to the initial DEM. Additionally, some artifacts are visible in the top left. Small details are visible, but the overall shape of the DEM seems to be distorted. For the AMSA ATM DEM created using the atmospherically compensated reflectance model, small details are generally visible. Some details are less pronounced compared to the good atmospheric conditions, e.g., the small crater in the western rim of the central basin. The DEMs for the AMSA ATM model with $\gamma =3\times 10^{-4}$ appear to be sharper in general (see Figure A7).

**Figure 12:**
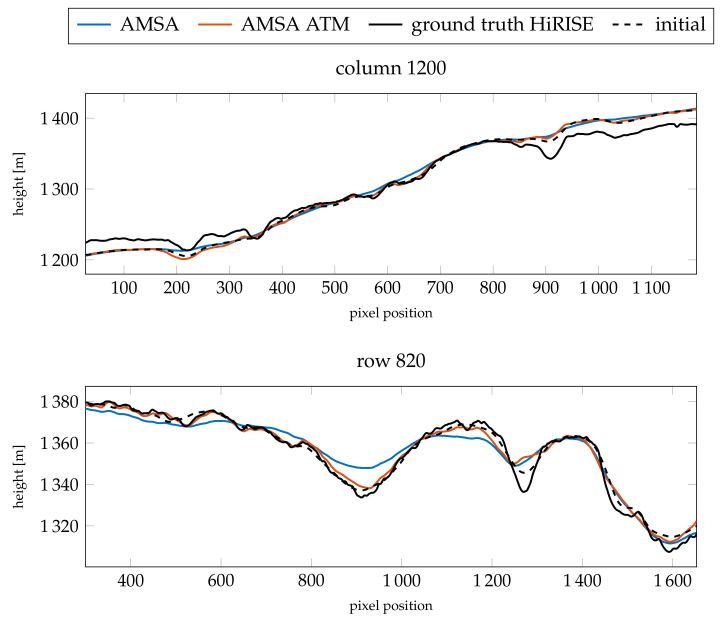
Resulting absolute heights compared for the two profiles investigated for image K13_058554_2232_XN_43N103W. The comparison is between the results for $\gamma =3\times 10^{-3}$ and the fixed optical depth of τ=0.61.

**Figure 13:**
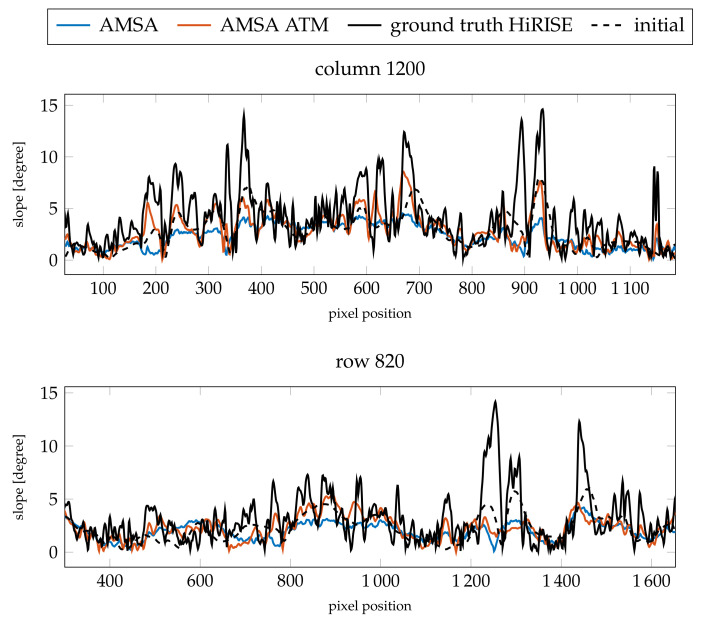
Resulting slopes compared for the two profiles investigated for image K13_058554_2232_XN_43N103W ($\gamma =3\times 10^{-3}$ and τ=0.61).

The profiles of the absolute heights (Figure 12) show that the AMSA DEM on a large scale has been flattened. According to the slopes (Figure 13), small details are still reconstructed, but are less pronounced. In contrast, the atmospheric model stays close to the initial DEM and small details are reconstructed. However, the small crater around pixel 1150 in row 820 is not reconstructed, and the large channel around pixel 1300 is not as deep as expected by the HiRISE ground truth. The slope at the eastern edge of the image is also too flat. Otherwise, a similar level of detail as the HiRISE ground truth can be achieved, as illustrated by the slopes. In column 1200, the main features are all represented in the SfS with atmospheric correction DEM.

### 3.4. Bad Atmospheric Conditions

Generally, the results for the bad atmospheric conditions are comparable to the previous image. The detailed results are listed in Table 7. Similar to the previous image, the AMSA model did diverge for $\gamma =3\times 10^{-4}$, and for $\gamma =3\times 10^{-3}$, the DEM has become too flat. For the model with an atmospheric correction, the errors are generally smaller for τ=0.59 compared to the fixed value of τ=0.94. This is in contrast to the previous image, where the fixed optical depth produced better results.

**Figure 14:**
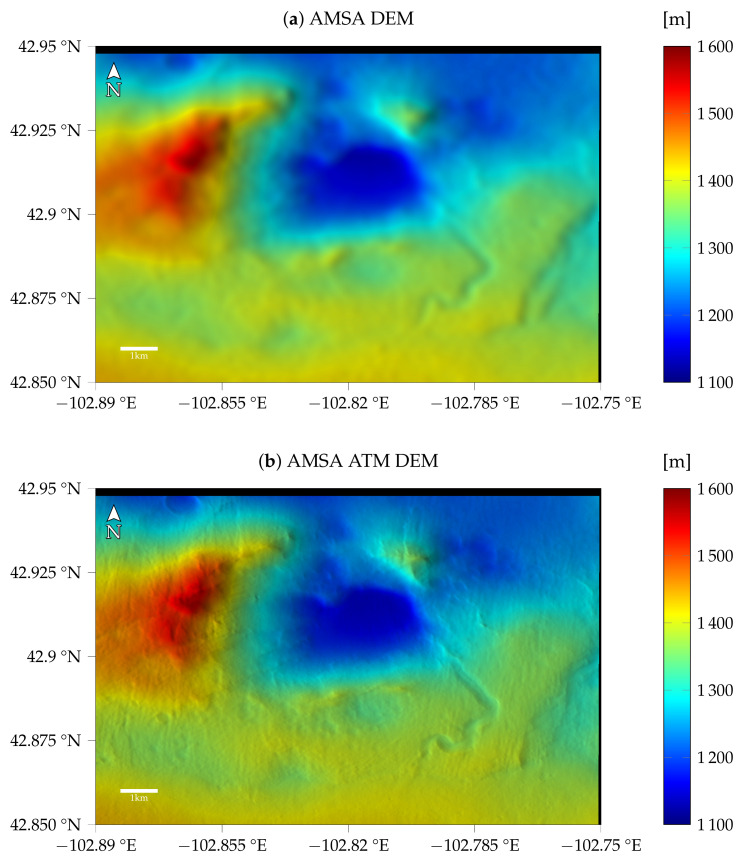
Results for image K13_058475_2232_XN_43N103W (**a**) without and (**b**) with atmospheric compensation. The results for $\gamma =3\times 10^{-4}$ and, in the case of atmospheric compensation, τ=0.59 are displayed.

Figure 14 shows the DEMs for $\gamma =3\times 10^{-4}$ and τ=0.59. The AMSA DEM looks like the initial DEM because it diverged, and the initial DEM was kept. Visually, the AMSA ATM DEM looks slightly sharper than the DEM with $\gamma =3\times 10^{-3}$ for the previous image. Small details are recovered.

**Figure 15:**
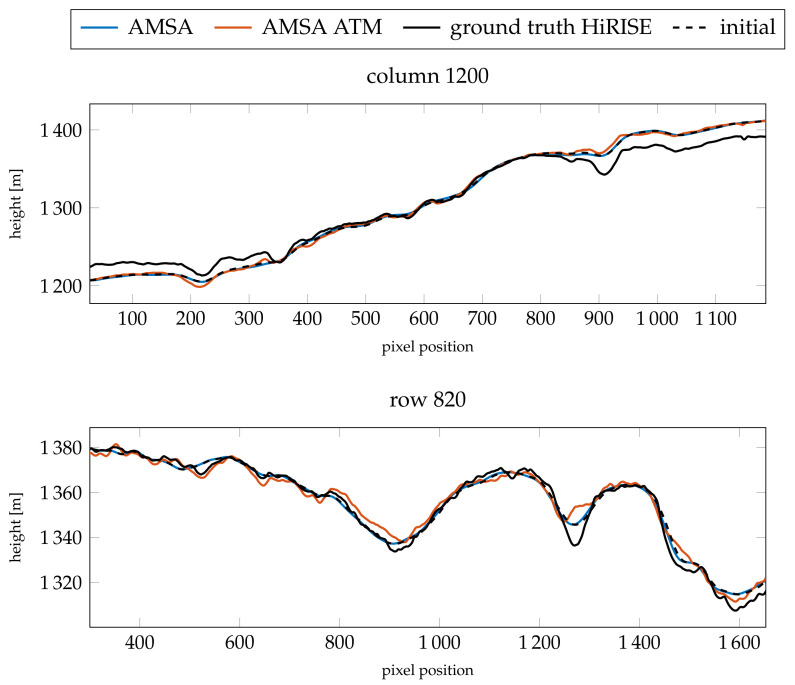
Resulting slopes compared for the two profiles investigated for image K13_058475_2232_XN_43N103W ($\gamma =3\times 10^{-4}$ and τ=0.59).

**Figure 16:**
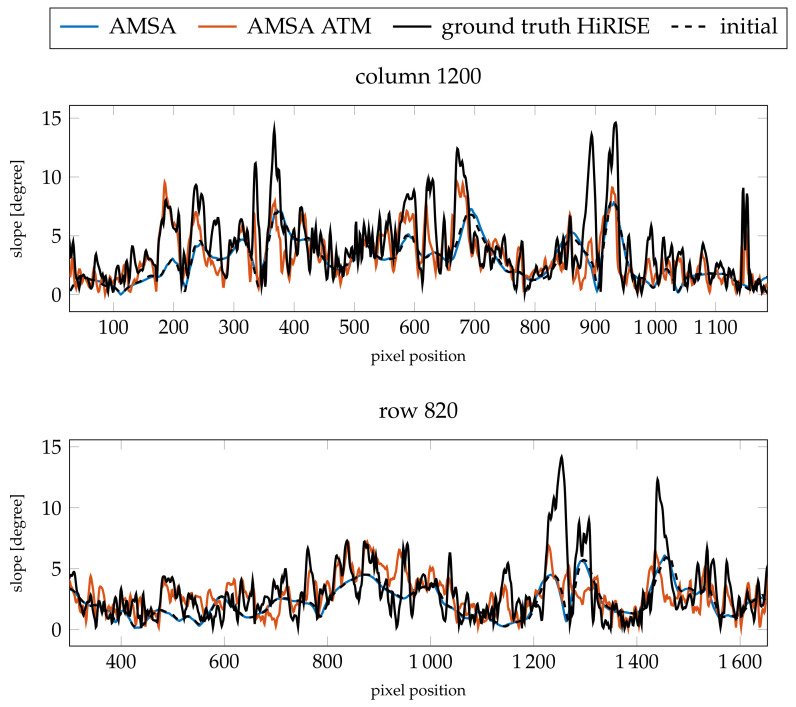
Resulting slopes compared for the two profiles investigated for image K13_058475_2232_XN_43N103W ($\gamma =3\times 10^{-4}$ and τ=0.59).

When investigating the absolute height profiles in Figure 15, it can be seen that some of the features are exaggerated, but the small crater, the channel and the slope in the east are again slightly too flat. Nonetheless, even for these poor conditions, a high level of detail can be reconstructed. The slopes for the profile in column 1200 (see Figure 16) are generally accurate for the atmospherically compensated SfS. Furthermore, the slopes in row 820 are accurate and detailed except for the high slopes in the areas mentioned above.

### 3.5. Discussion

Under good atmospheric conditions, i.e., small values of τ, the atmospheric correction model does not have a perceivable effect. This behavior is illustrated by the fact that the DEMs constructed with the AMSA and the AMSA ATM model, respectively, are largely identical for the first image.

There is a discrepancy between the HiRISE stereo and the CTX stereo DEM, which served as an initialization and constraint for our SfS scheme. Because these low frequency differences cannot be corrected by the SfS, this effect propagates to the results as well. Both DEMs are created with the same method using the Ames stereo pipeline, but are derived from different data sets. This mismatch could be due to several reasons. Kirk et al. [65] evaluated the differences of stereo DEMs based on HiRISE and CTX images and found that vertical precision depends on the image noise as well as illumination and terrain roughness for different image resolutions. We have thoroughly co-registered the DEMs, but there might still be small differences that could contribute to the deviations of absolute heights. Another factor that might influence the vertical accuracy of the stereo DEMs are the SPICE-kernels and the map projection step in ISIS. For example, small inaccuracies in the positioning of the camera can lead to systematically different height estimates.

The behavior of our DEM construction system changes for moderate atmospheric conditions. The reconstruction algorithm using the AMSA model without atmospheric correction does not converge. In contrast, the AMSA ATM model recovers a level of topographic detail that is similar to the HiRISE ground truth DEM. We run the procedure with a fixed τ taken from Montabone et al. [34] and estimate τ directly from the scene. The estimated τ is slightly smaller compared to the value from Montabone et al. [34], but is still consistent with medium atmospheric conditions. The quality of the resulting DEMs is almost the same given the RMSEs and the visual inspection. This highlights the robustness of the procedure against slight variations of the optical depth. Rather, the selection of γ appears to have a larger impact on the level of detail recovered.

Under bad atmospheric conditions with further increasing optical depth, the AMSA model without atmospheric correction does not lead to a meaningful solution. Again, the AMSA ATM model yields a DEM that still shows a remarkable level of detail given the challenging atmospheric conditions. The optical depth estimated by the AMSA ATM model (τ=0.59) is smaller than the value of Montabone et al. [34] (τ=0.94). However, the optical depth directly estimated from the image data produces a measurably better DEM for this image. In general, the AMSA ATM model can estimate the atmospheric parameters directly from the image data without the need for external measurements.

These results have general implications for intensity-based Martian 3D-reconstruction. A reconstruction procedure without explicit atmospheric modeling might be a viable approximation for excellent atmospheric conditions, as the influence of the atmosphere can largely be neglected. For increasing optical depth, atmospheric modeling is necessary. We found that the atmospheric parameters can be directly estimated from the scene. Some recent 3D-reconstruction approaches employ machine learning (ML), which is entirely data-driven. Consequently, the ML model has to learn the entire relationship between image intensity and heights (or slopes), which also encompasses atmospheric influence. Our study implies that the ML practitioner should either limit the training and application to images taken only under good atmospheric conditions, or aim for a dataset that adequately captures imagery with various optical depths.

The proposed combined model of surface reflectance and atmosphere has proven to be suitable for reconstructing the surface of Mars and bears several advantages compared to earlier methods. The model parameters have a physical representation and can be interpreted intuitively. Surface reflectance is modeled with the widely used and state-of-the-art Hapke model [43], in contrast to simpler models such as Lambert or Oren–Nayar (e.g., [13,14,16]) employed in earlier approaches. The single scattering albedo is not assumed to be constant, but rather is updated throughout the SfS scheme accounting for local albedo variations present on Mars. The atmospheric parameters can be estimated directly from the image data instead of using complicated retrieval algorithms relying on external data sets.

## 4. Conclusions

In this study, we describe a framework for the Shape from Shading based construction of DEMs of the Martian surface using CTX images. Our method is based on the Hapke AMSA model [43], which is enhanced by a correction of atmospheric effects that takes into account the optical depth of the atmosphere, the influence of surface illumination by the bright sky and the path-scattered component leading to a largely uniform intensity background (AMSA ATM model). The atmospheric parameters can be estimated from the image data alone using Bayesian optimization without the need to refer to external data sources. The optical depth values estimated by our method are mostly comparable with those independently measured in Montabone et al. [33] and Montabone et al. [34]. For a clear, dust-free atmosphere, the difference between the DEMs constructed with the AMSA ATM model and with the simple AMSA model is negligible. For a more dust-laden atmosphere leading to moderate or bad atmospheric conditions, the reconstruction with the AMSA model without atmospheric correction does not show a reasonable convergence behavior, so no well-defined DEM can be obtained. In contrast, the AMSA ATM model yields a high-resolution DEM, which shows a similar level of detail as the image for all considered atmospheric conditions. Consequently, the developed AMSA ATM model allows for the construction of high-resolution DEMs across a broad range of atmospheric conditions on Mars.

## Figures and Tables

**Table 1:** All physical and geometric parameters of the reflectance model and atmospheric model. The shadow hiding opposition effect and the coherent backscatter opposition effect are abbreviated as SHOE and CBOE, respectively.

Group	Parameter	Description	Method			Resolution		Values	
			A Priori	One-Shot	Iterative	Constant	Pixelwise	Fixed	Range
Geometrics	$\mu_{0}s$	Cosine of sun zenith	✓			✓			
	$\mu_{s}$	Cosine of orbiter zenith	✓			✓			
	$\theta_{i}$	Incidence angle			✓		✓		
	$\theta_{e}$	Emission angle			✓		✓		
	g	Phase angle			✓		✓		
Material	*w*	Single-scattering albedo			✓		✓		0.35 ≤ *w* ≤ 0.95
	*b*	Backscattering p(g)	✓			✓		0.12	
	*c*	Asymmetry p(g)	✓			✓		0.6	
	$B_{S0}$	SHOE strength	✓			✓		3.1	
	$h_{s}$	SHOE shape	✓			✓		0.11	
	$B_{C0}$	CBOE strength	✓			✓		0	
	$h_{c}$	CBOE shape	✓			✓		0	
	$\bar{\theta }$	Average roughness	✓			✓		$0^{\circ }$	
Atmospheric	τ	Optical depth		✓		✓			0.1≤τ≤3.0
	ζ	contribution $r_{\mathrm{hd}}$		✓		✓			0 ≤ζ≤∼ 0.2
	χ	Background illum.		✓		✓			0 ≤χ≤∼ 0.02

**Table 2:** Parameters for the regularization terms of the Shape from Shading (SfS) scheme.

SfS Parameter	Regularization Term	Value
γ	Integrability Error	Chosen individually
$\tau_{SfS}$	Absolute Heights Error	$1\times 10^{-2}$
δ	Gradients Error	$1\times 10^{-2}$
σ	Width of the Gaussian low pass	10 pix

**Table 3:** CTX images used in this work. Dust Optical Depth (DOD) in the visible wavelength range from Montabone et al. [33] and Montabone et al. [34]. Region of interest: latitude 42.850° N to 42.95° N and longitude −102.89° E to −102.75° E. G20_025904_2209_XN_40N102W and G20_025970_2217_XN_41N102W were used for generating the initial stereo DEM. G20_025904_2209_XN_40N102W was not used for the SfS generation.

Image ID	MY	SOL	DOD	Phase Angle
G20_025904_2209_XN_40N102W	31	141	0.267	47.25°
G20_025970_2217_XN_41N102W	31	146	0.160	31.45°
K13_058554_2232_XN_43N103W	34	611	0.615	80.85°
K13_058475_2232_XN_43N103W	34	607	0.945	66.94°

**Table 4:** Parameters obtained by Bayesian optimization in MATLAB^®^ based on sample profiles in the three images with free and fixed values of τ. The fixed values are taken from Montabone et al. [33] and Montabone et al. [34]. For G20_025970_2217_XN_41N102W, τ is fixed and all other parameters are free. The resulting mean albedo $w_{0}$ from that image is 0.81, and is consequently used as a fixed mean albedo for the other images.

Product ID	*w*	τ	ζ	χ	RMSE
G20_025970_2217_XN_41N102W (τ fixed)	0.81	0.16	0.00198	0.0043	0.0024
K13_058554_2232_XN_43N103W (τ free)	0.81	0.49	0.09983	0.01187	0.00084
K13_058554_2232_XN_43N103W (τ fixed)	0.81	0.61	0.099	0.0121	0.00062
K13_058475_2232_XN_43N103W (τ free)	0.81	0.59	0.099	0.0114	0.00079
K13_058475_2232_XN_43N103W (τ fixed)	0.81	0.94	0.1159	0.0199	0.00106

**Table 5:** Comparison of the Root Mean Square Errors (RMSEs) for the examined lines in the DEMs based on image G20_025970_2217_XN_41N102W. The HiRISE DEM is the reference DEM to which the other DEMs are compared to. The initial DEM is the CTX stereo DEM, which is refined with the AMSA or AMSA ATM model.

Row/Column	Model	Optical Depth	γ	RMSE Heights	RMSE Slopes
Row 820	Initial	-	-	2.704	2.111
	AMSA	-	$1\times 10^{-3}$	3.563	1.947
	AMSA ATM	0.16	$1\times 10^{-3}$	3.273	1.934
Column 1200	Initial	-	-	12.430	2.568
	AMSA	-	$1\times 10^{-3}$	12.230	1.819
	AMSA ATM	0.16	$1\times 10^{-3}$	12.184	1.787

**Table 6:** Comparison of the Root Mean Square Errors (RMSEs) for the examined lines in the DEMs based on image K13_058554_2232_XN_43N103W. $\gamma =3\times 10^{-3}$ is the best value for the AMSA model and $\gamma =3\times 10^{-4}$ is the best value for the AMSA ATM model determined by the heuristic described in Section 2.3.

Row/Column	Model	Optical Depth	γ	RMSE Heights	RMSE Slopes
Row 820	Initial	-	-	2.704	2.111
	AMSA	-	$3\times 10^{-3}$	5.370	2.499
	AMSA	-	$3\times 10^{-4}$	2.668	2.144
	AMSA ATM	0.49	$3\times 10^{-3}$	3.978	2.349
	AMSA ATM	0.61	$3\times 10^{-3}$	3.606	2.317
	AMSA ATM	0.49	$3\times 10^{-4}$	4.229	2.107
	AMSA ATM	0.61	$3\times 10^{-4}$	4.009	2.094
Column 1200	Initial	-	-	12.430	2.568
	AMSA	-	$3\times 10^{-3}$	13.308	3.084
	AMSA	-	$3\times 10^{-4}$	12.551	2.616
	AMSA ATM	0.49	$3\times 10^{-3}$	13.337	2.508
	AMSA ATM	0.61	$3\times 10^{-3}$	13.227	2.429
	AMSA ATM	0.49	$3\times 10^{-4}$	13.088	2.269
	AMSA ATM	0.61	$3\times 10^{-4}$	13.096	2.171

**Table 7:** Comparison of the Root Mean Square Errors (RMSEs) for the examined lines in the DEMs based on image K13_058475_2232_XN_43N103W. $\gamma =3\times 10^{-3}$ is the best value for the AMSA model and $\gamma =3\times 10^{-4}$ is the best value for the AMSA ATM model determined by the heuristic described in Section 2.3.

Row/Column	Model	Optical Depth	γ	RMSE Heights	RMSE Slopes
Row 820	Initial	-	-	2.704	2.111
	AMSA	-	$3\times 10^{-3}$	6.798	2.533
	AMSA	-	$3\times 10^{-4}$	2.659	2.143
	AMSA ATM	0.59	$3\times 10^{-3}$	2.882	2.182
	AMSA ATM	0.94	$3\times 10^{-3}$	2.709	2.129
	AMSA ATM	0.59	$3\times 10^{-4}$	3.692	2.040
	AMSA ATM	0.94	$3\times 10^{-4}$	4.893	2.523
Column 1200	Initial	-	-	12.430	2.568
	AMSA	-	$3\times 10^{-3}$	13.201	3.153
	AMSA	-	$3\times 10^{-4}$	12.561	2.619
	AMSA ATM	0.59	$3\times 10^{-3}$	12.774	2.356
	AMSA ATM	0.94	$3\times 10^{-3}$	12.641	2.454
	AMSA ATM	0.59	$3\times 10^{-4}$	13.068	2.075
	AMSA ATM	0.94	$3\times 10^{-4}$	13.640	2.449

**Figure A2:**
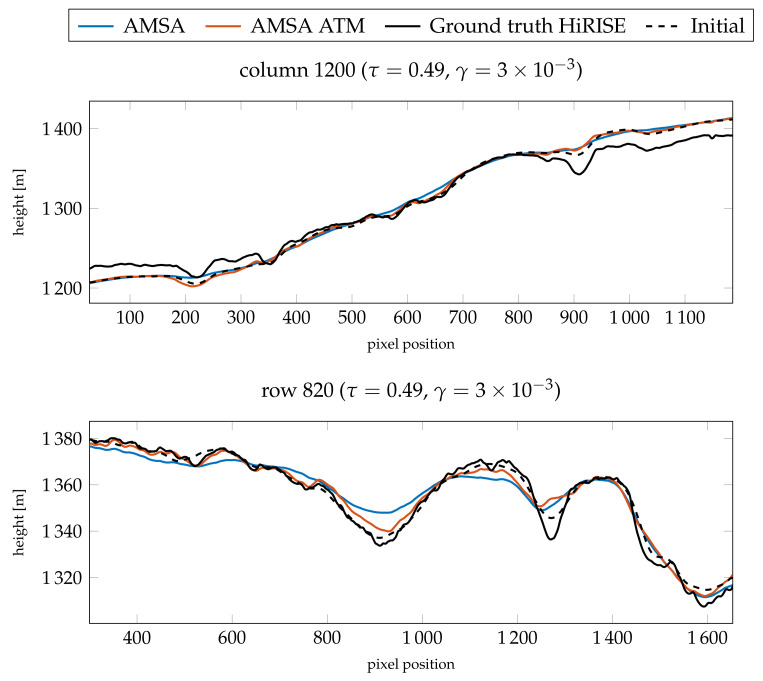

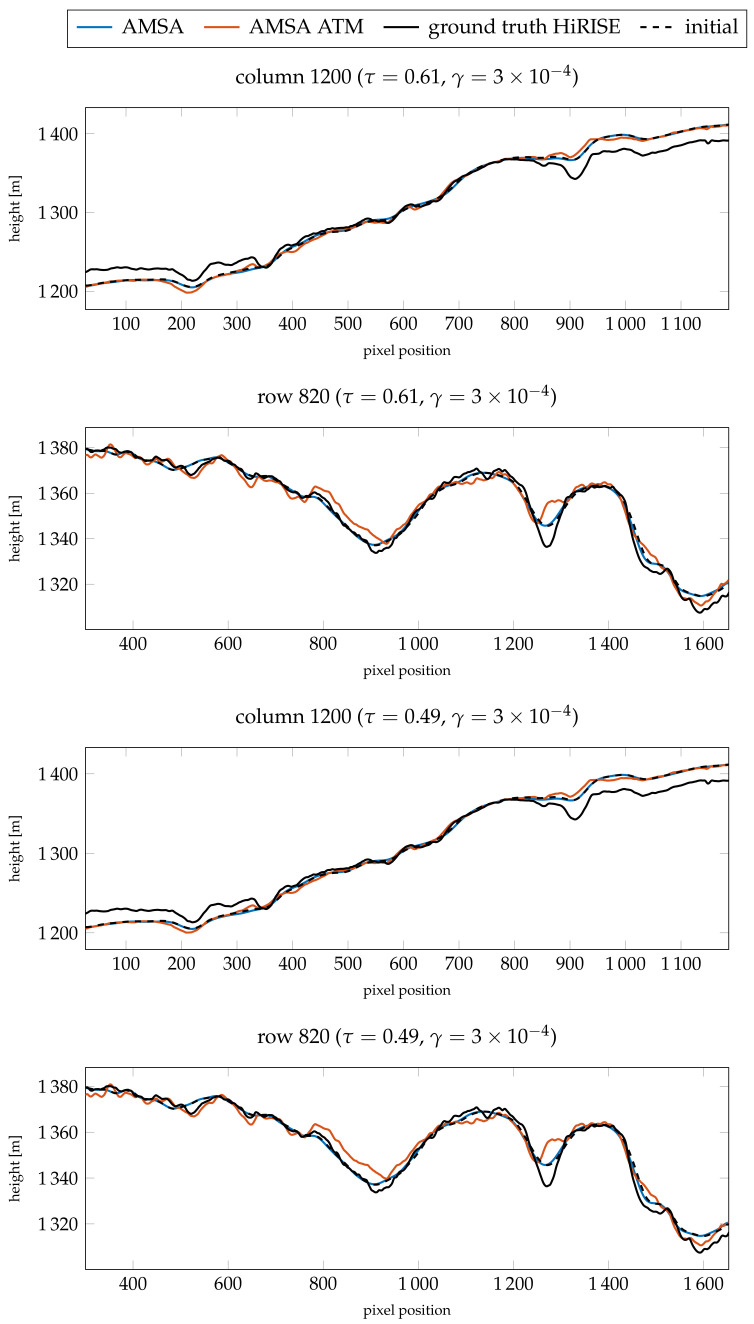
Absolute heights for image K13_058554_2232_XN_43N103W (medium atmospheric conditions) not displayed in the main text.

**Figure A3:**
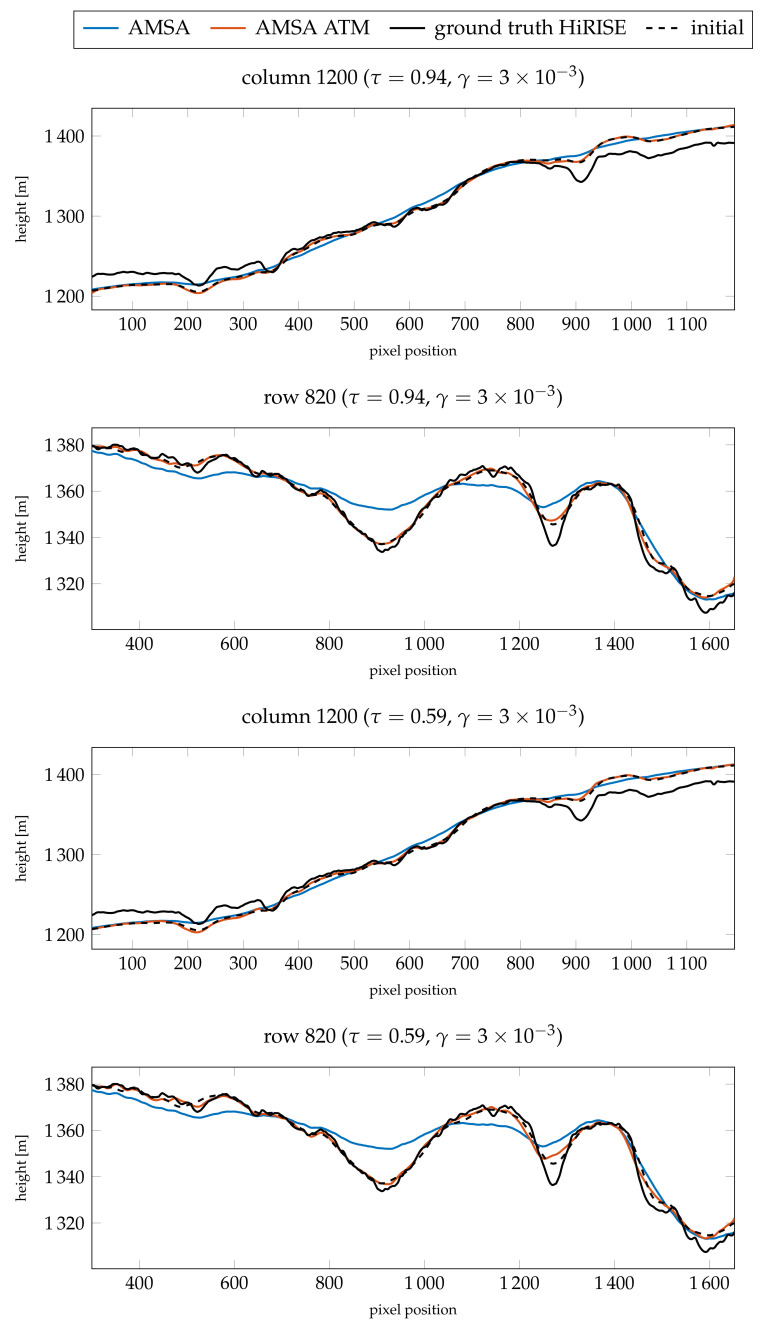

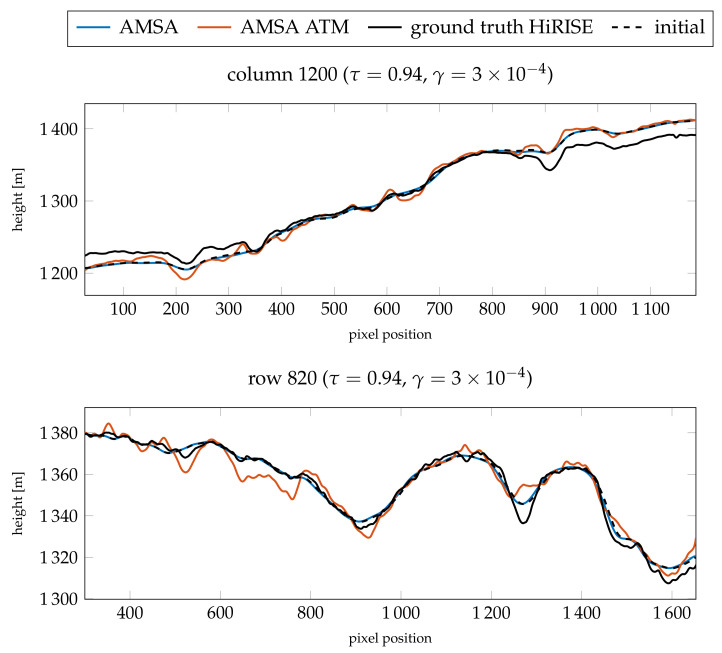
Absolute heights for image K13_058475_2232_XN_43N103W (bad atmospheric conditions) not displayed in the main text.

**Figure A4:**
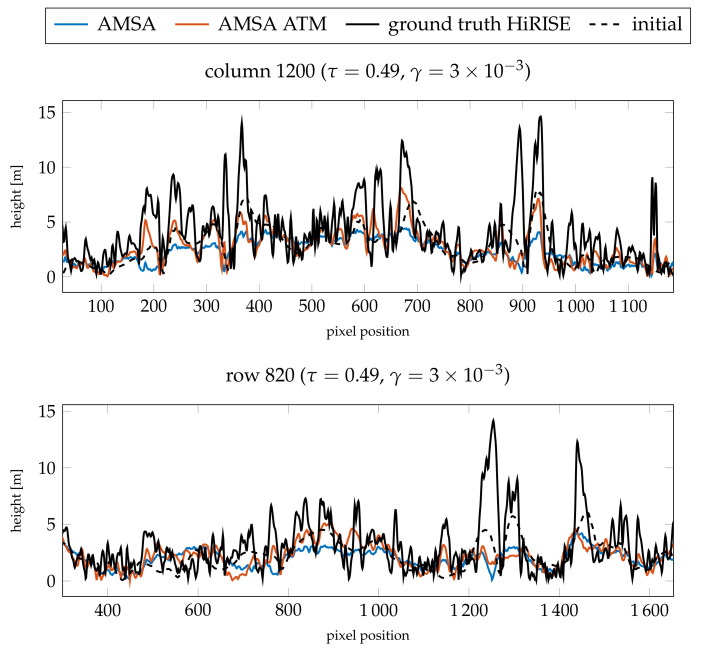

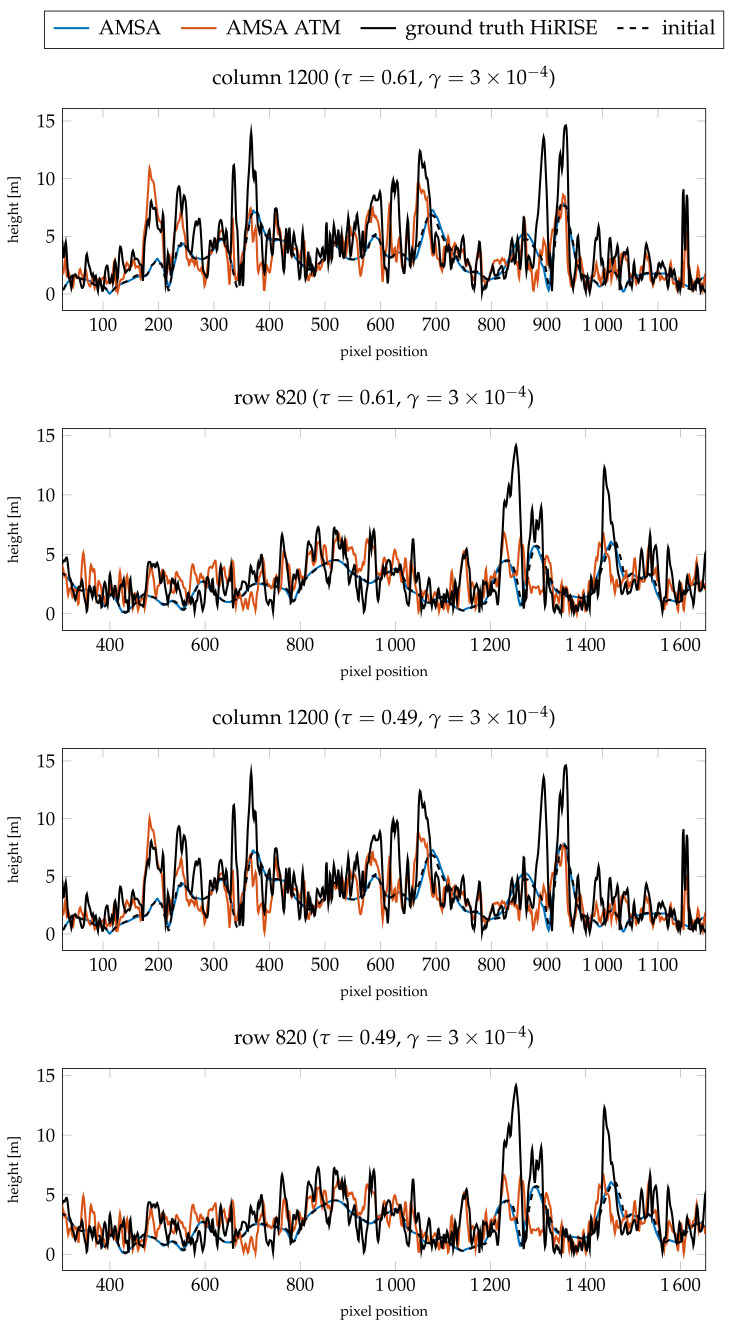
Slopes for image K13_058554_2232_XN_43N103W (medium atmospheric conditions) not displayed in the main text.

**Figure A5:**
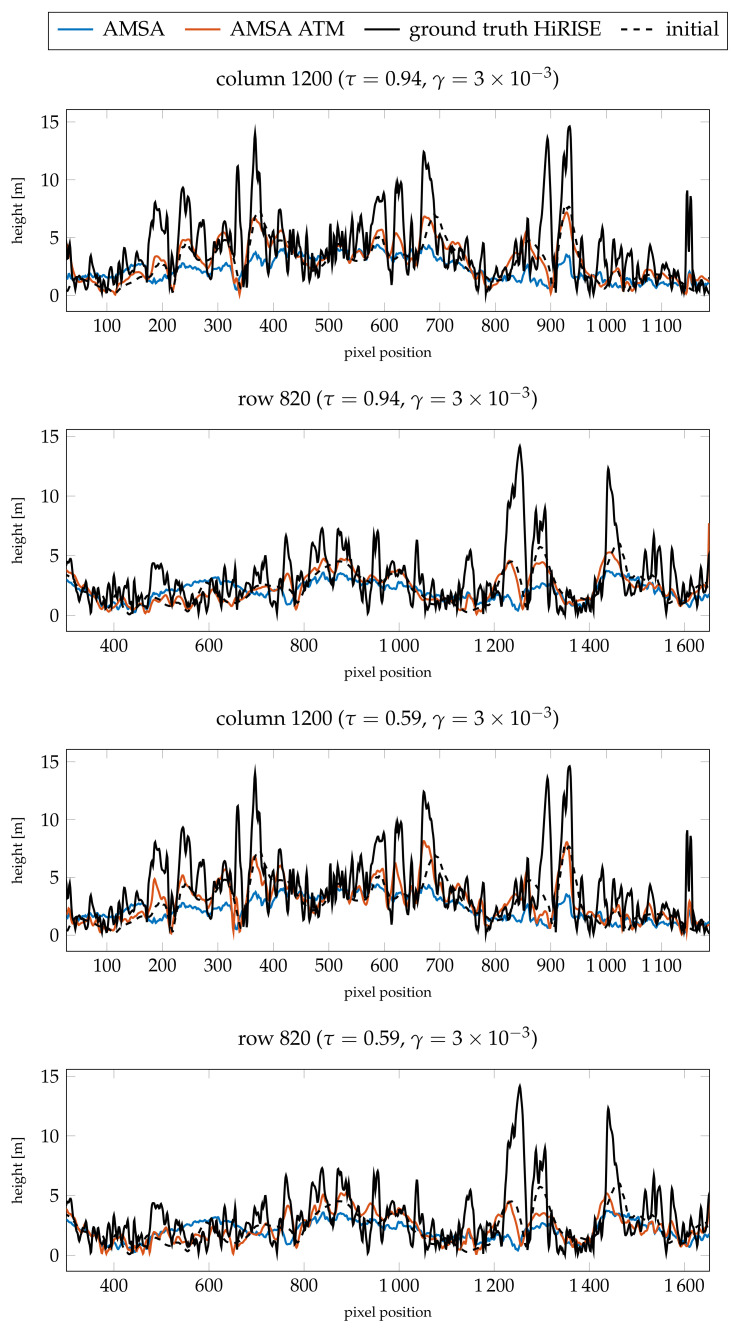

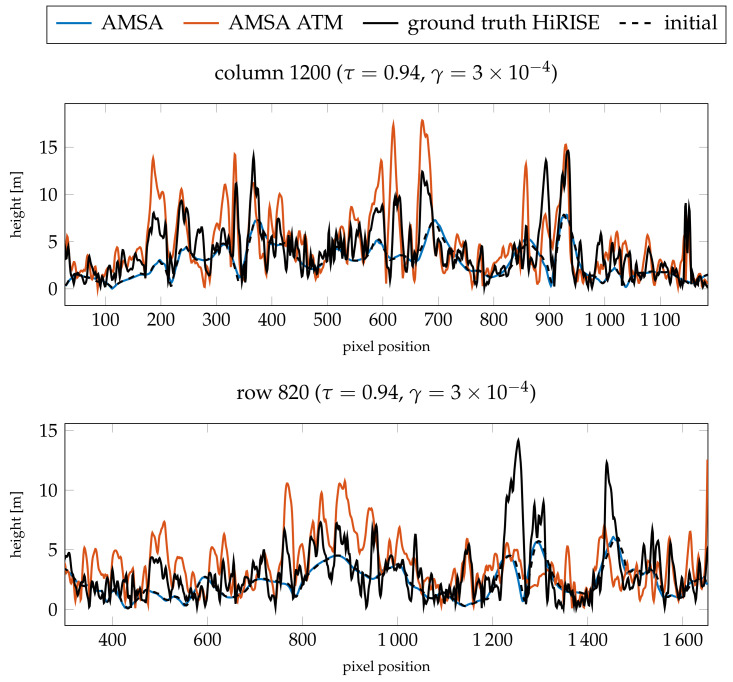
Slopes for image K13_058475_2232_XN_43N103W (bad atmospheric conditions) not displayed in the main text.

**Figure A6:**
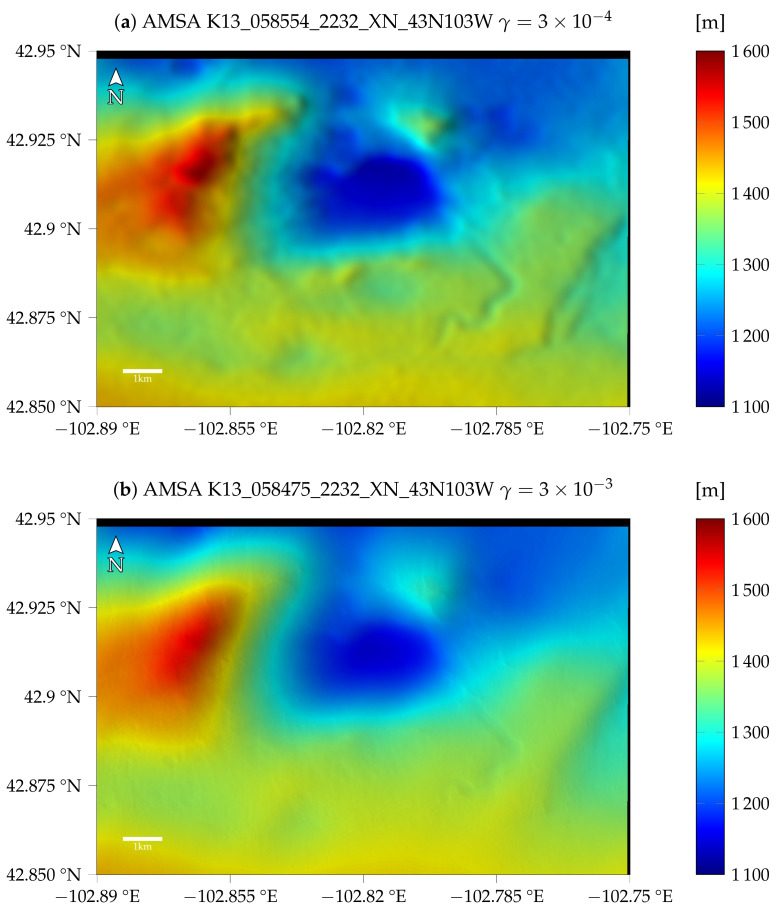
Results for images K13_058554_2232_XN_43N103W and K13_058475_2232_XN_43N103W for the simple AMSA model without atmospheric compensation.

**Figure A8:**
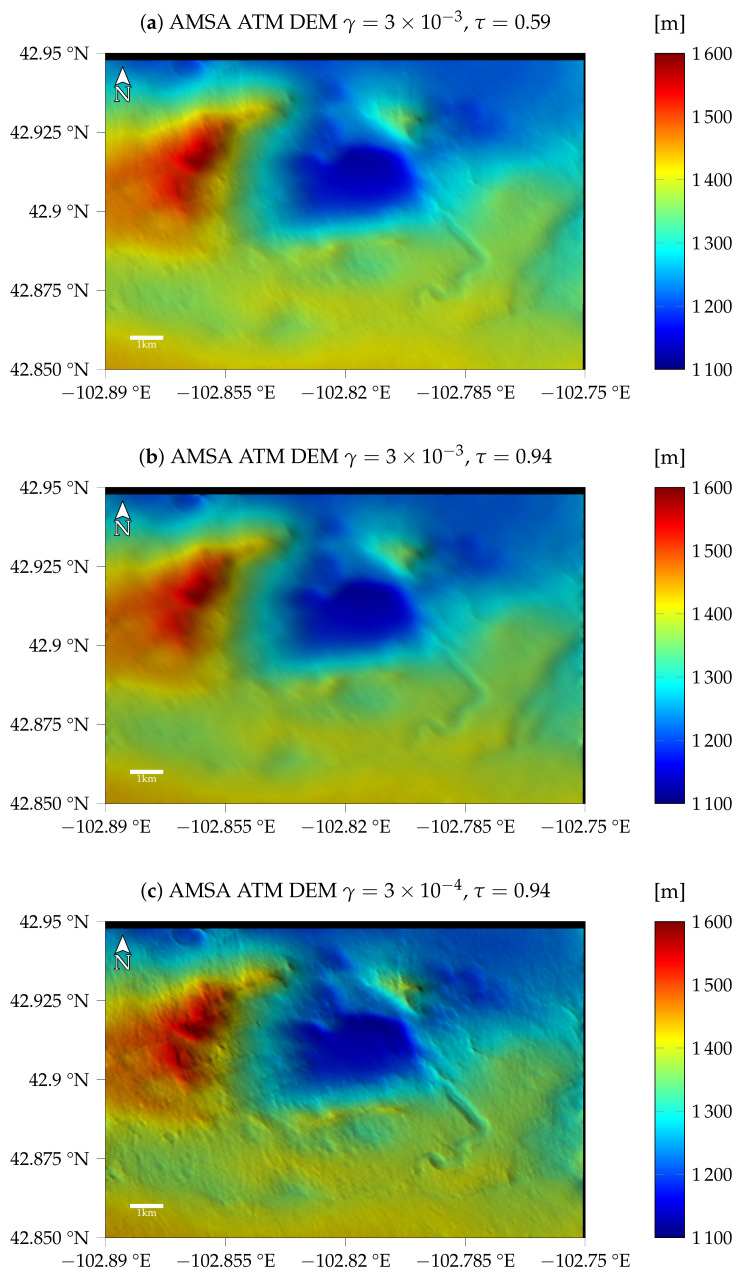
Color-coded and shaded DEMs created with the atmospherically compensated method not displayed in the main text of the manuscript for image K13_058475_2232_XN_43N103W (bad atmospheric conditions).

## Data Availability

Not applicable.

## Appendix A

Here we briefly review the method given by Grumpe and Wöhler [1]. The normal vector of a surface facet is defined as

(A1) $$n=\frac{1}{\sqrt{p^{2}+q^{2}+1}}\left(\begin{array}{c} -p\\ -q\\ 1 \end{array}\right)$$

where *p* and *q* are the partial derivatives of the DEM *z* with respect to the x and y directions, i.e., the rows and the columns of an image:

(A2) $$p=\frac{\partial z}{\partial x}\;\mathrm{and}\;q=\frac{\partial z}{\partial y}.$$

The objective function can be formulated as a weighted sum of several error terms:

(A3) $$E_{\mathrm{total}}=E_{I}+\lambda E_{\mathrm{int}}+\tau_{a}E_{\mathrm{absolute}}+\delta E_{\mathrm{gradient}}.$$

$E_{\mathrm{total}}$ primarily penalizes the deviation between modeled reflectance using the estimated DEM and the reflectance image *I* captured by the orbiter:

(A4) $$E_{I}=\frac{1}{2}\int_{x}\int_{y}{\left(R\left(p,q\right)-I\right)}^{2}dxdy.$$

Further, the integrability of the estimated vector field is enforced by requesting

(A5) $$E_{\mathrm{int}}=\frac{1}{2}\int_{x}\int_{y}{\left(\frac{\partial z}{\partial x}-p\right)}^{2}+{\left(\frac{\partial z}{\partial y}-q\right)}^{2}dxdy.$$

The absolute error term

(A6) $$E_{\mathrm{absolute}}=\frac{1}{2}\int_{x}\int_{y}{\left(f_{\mathrm{Gauss}}\left(z\right)-f_{\mathrm{Gauss}}\left(z_{\mathrm{DTM}}\right)\right)}^{2}dxdy$$

elastically pins the DEM to the absolute heights given by a low-resolution constraint DEM. Finally, the relative depth error term

(A7) $$\begin{array}{cc} E_{\mathrm{gradient}}= & \frac{1}{2}\int_{x}\int_{y}{\left(f_{\mathrm{lp}}\left(p\right)-f_{\mathrm{lp}}\left(\frac{\partial z_{\mathrm{DEM}}}{\partial x}\right)\right)}^{2}\\ \; & +{\left(f_{\mathrm{lp}}\left(q\right)-f_{\mathrm{lp}}\left(\frac{\partial z_{\mathrm{DEM}}}{\partial y}\right)\right)}^{2}dxdy \end{array}$$

imposes a further constraint that the low frequency component of the gradients of the initial DEM align with the low frequency component of the estimated gradients.

To obtain the surface *z*, the error $E_{\mathrm{total}}$ has to be minimized. This is done with an iterative relaxation approach that means that, alternately, either the variables $p_{u,v}^{n+1}$ and $q_{u,v}^{n+1}$ are updated, assuming that the solution $z_{u,v}^{n}$ is known, or the solution $z_{u,v}^{n+1}$ is updated given $p_{u,v}^{n}$ and $q_{u,v}^{n}$. If $z_{u,v}^{n}$ is given at time step *n*, the terms $E_{I}$, $E_{\mathrm{int}}$, and $E_{\mathrm{gradient}}$ are the only ones that depend on *p* and *q*, which can be optimized by deriving the error terms and solving for *p* and *q*:

(A8) $$\begin{array}{cc} p_{u,v}^{\left(n+1\right)}= & \frac{\partial z}{\partial x}{|}_{u,v}^{\left(n\right)}\\ \; & -\frac{1}{\gamma }\left(R\left(\frac{\partial z}{\partial x}{|}_{u,v}^{\left(n\right)},\frac{\partial z}{\partial y}{|}_{u,v}^{\left(n\right)}\right)-I_{u,v}\right)\frac{\partial R}{\partial p}{|}_{\frac{\partial z}{\partial x}{|}_{u,v}^{\left(n\right)},\frac{\partial z}{\partial y}{|}_{u,v}^{\left(n\right)}}^{\left(n\right)}\\ \; & +\frac{\delta }{\gamma }\left(g_{\sigma_{grad}}\circ g_{\sigma_{grad}}*\left(\frac{\partial z}{\partial x}-\frac{\partial z_{DEM}}{\partial x}\right)\right) \end{array}$$

(A9) $$\begin{array}{cc} q_{u,v}^{\left(n+1\right)}= & \frac{\partial z}{\partial y}{|}_{u,v}^{\left(n\right)}\\ \; & -\frac{1}{\gamma }\left(R\left(\frac{\partial z}{\partial x}{|}_{u,v}^{\left(n\right)},\frac{\partial z}{\partial y}{|}_{u,v}^{\left(n\right)}\right)-I_{u,v}\right)\frac{\partial R}{\partial p}{|}_{\frac{\partial z}{\partial x}{|}_{u,v}^{\left(n\right)},\frac{\partial z}{\partial y}{|}_{u,v}^{\left(n\right)}}^{\left(n\right)}\\ \; & +\frac{\delta }{\gamma }\left(g_{\sigma_{grad}}\circ g_{\sigma_{grad}}*\left(\frac{\partial z}{\partial y}-\frac{\partial z_{DEM}}{\partial y}\right)\right) \end{array}$$

If *p* and *q* are known at time step *n*, the terms $E_{\mathrm{int}}$ and $E_{\mathrm{absolute}}$ depend on the variable *z*. To find the optimal surface shape, calculus of variations must be employed, which yields a polynomial

(A10) $$c_{2}z^{2}+c_{1}z+c_{0}=0$$

with

(A11) $$\begin{array}{cc} c_{0}= & -\hat{\tau }\left(f_{-0}\left(z\right)-f_{DTM}\left(z_{DTM}\right)\right)\;\cdot \;\frac{h_{cx}^{2}z_{h}+h_{cy}^{2}z_{v}}{h_{cx}^{2}h_{cy}^{2}}\\ \; & \;\cdot \;\left(\frac{z_{x}\left(\frac{F_{l}}{2h_{r}}z_{2r}-\frac{F_{r}}{2h_{l}}z_{2l}+f_{-h}\left(z_{x}\right)\right)}{z_{x}^{2}+z_{y}^{2}}\right.\\ \; & +\left.\frac{+z_{y}\left(\frac{F_{d}}{2h_{u}}z_{2u}-\frac{F_{u}}{2h_{d}}z_{2d}+f_{-v}\left(z_{y}\right)\right)}{z_{x}^{2}+z_{y}^{2}}\right)\\ \; & -\left(p_{x}+q_{y}\right) \end{array}$$

(A12) $$\begin{array}{cc} c_{1}= & -\hat{\tau }\left(f_{-0}\left(z\right)-f_{DTM}\left(z_{DTM}\right)\right)\\ \; & \;\cdot \;\frac{z_{x}\left(\frac{F_{r}}{2h_{l}}-\frac{F_{l}}{2h_{r}}\right)+z_{y}\left(\frac{F_{u}}{2h_{d}}-\frac{F_{d}}{2h_{u}}\right)}{z_{x}^{2}+z_{y}^{2}}\\ \; & -\hat{\tau }F_{c}\frac{z_{x}\left(\frac{F_{l}}{2h_{r}}z_{2r}-\frac{F_{r}}{2h_{l}}z_{2l}+f_{-h}\left(z_{x}\right)\right)}{z_{x}^{2}+z_{y}^{2}}\\ \; & -\hat{\tau }F_{c}\frac{z_{y}\left(\frac{F_{d}}{2h_{u}}z_{2u}-\frac{F_{u}}{2h_{d}}z_{2d}+f_{-v}\left(z_{y}\right)\right)}{z_{x}^{2}+z_{y}^{2}}\\ \; & -\frac{2(h_{cx}^{2}+h_{cy}^{2})}{h_{cx}^{2}h_{cy}^{2}} \end{array}$$

(A13) $$c_{2}=-\hat{\tau }F_{c}\frac{z_{x}\left(\frac{F_{r}}{2h_{l}}-\frac{F_{l}}{2h_{r}}\right)+z_{y}\left(\frac{F_{u}}{2h_{d}}-\frac{F_{d}}{2h_{u}}\right)}{z_{x}^{2}+z_{y}^{2}}.$$

The polynomial has two roots. The root closer to the initial DEM gives the final result for *z*.
